# Maternal RSVpreF Immunisation Against Infant RSV Hospitalisation: Nationwide Population-Based Effectiveness and Durability Study

**DOI:** 10.1016/j.lanepe.2026.101756

**Published:** 2026-06-20

**Authors:** Marie-Joelle Jabagi, Marion Bertrand, Amélie Gabet, Ludovic Tréluyer, Epiphane Kolla, Antoine Rachas, Valérie Olié, Mahmoud Zureik

**Affiliations:** a French National Agency for Medicines and Health Products Safety and French National Health Insurance (EPI-PHARE), Saint-Denis, France; b Department of Neonatal Intensive Care, FHU Prem'Impact, Cochin-Port Royal Hospital, Assistance Publique-Hôpitaux de Paris, Paris Cité University, 75014, Paris, France; c Anti-Infective Evasion and Pharmacoepidemiology, INSERM, Center for Research in Epidemiology and Population Health, University of Paris-Saclay, Montigny le Bretonneux, France

**Keywords:** Respiratory syncytial virus, Maternal vaccination, RSVpreF, Hospitalisation, Immunisation

## Abstract

**Background:**

Population-level real-world evidence effectiveness on the newly introduced maternal RSVpreF vaccine remains limited. We estimated effectiveness and durability of protection against RSV-associated hospitalisation in infants.

**Methods:**

Using the French National Health Data System, we conducted a nationwide study emulating a target trial among infants born between Sept 1 and Dec 31, 2024 in France. Infants born to mothers vaccinated with RSVpreF during pregnancy were matched 1:1 with unimmunised infants on exact date of birth, gestational age, sex, and deprivation status. Follow-up started at birth and continued until Feb 28, 2025. Inverse probability weighting was applied to balance measured covariates. The primary outcome was hospitalisation for RSV-associated lower respiratory tract infection (RSV-LRTI). Secondary outcomes included paediatric intensive care unit admission, oxygen therapy, and ventilatory support. Hazard ratios were estimated using Cox models, and vaccine effectiveness calculated as (1-HR) × 100.

**Findings:**

Among 31,356 infants included in the matched cohort (15,678 per group; median follow-up 86 days [IQR 73–101]), 693 RSV-LRTI hospitalisations occurred, including 216 (1·4%) among immunised infants and 477 (3·0%) among unimmunised. Maternal RSVpreF immunisation was associated with a reduced risk of RSV-LRTI hospitalisation (HR 0·50, 95% CI 0·45–0·55), corresponding to an effectiveness of 50% (95% CI 45–55). Protection was highest during days 0–14 after birth (66%, 95% CI 58–73) and declined thereafter to 38% (95% CI 19–52) during days 60–75. Reductions were also observed for severe outcomes, including RSV-LRTI requiring paediatric intensive care unit admission (74 (0·5%) vs 158 (1·0%); effectiveness 51%, 95% CI 43–57), oxygen therapy (59 (0·4%) vs 136 (0·9%); effectiveness 54%, 95% CI 46–60), and ventilatory support (65 (0·4%) vs 137 (0·9%); effectiveness 53%, 95% CI 45–59).

**Interpretation:**

Maternal RSVpreF immunisation reduced RSV-associated hospitalisation in infants, with protection strongest early in life and declining thereafter.

**Funding:**

No funding.

**Research in context**

**Evidence before this study**

We searched PubMed for studies published between Jan 1, 2022 and Feb 1, 2026 using the terms “respiratory syncytial virus”, “maternal vaccination”, “RSVpreF”, and “hospitalisation”, with no language restrictions. Respiratory syncytial virus (RSV) is the leading cause of lower respiratory tract infection hospitalisation (RSV-LRTI) in infants worldwide. In the phase 3 MATISSE trial, maternal RSVpreF vaccination reduced RSV-associated hospitalisation by 67·7% within 90 days and 56·8% within 180 days after birth. Observational studies from the United Kingdom reported effectiveness estimates ranging from 58% (95% CI 28–75) to 72% (48–85) depending on the interval between vaccination and delivery, while studies from Scotland, Argentina, and the United States reported effectiveness estimates of 82% (75–87), 79% (62–88), and 70% (37–86), respectively, against RSV-associated hospitalisation in early infancy. These studies used test-negative or case–control designs and many were conducted in selected regions or during early phases of programme rollout. Evidence remains limited regarding protection against severe outcomes, durability of protection across the first RSV season, and how effectiveness varies according to vaccination-to-delivery intervals, gestational timing of vaccination, maternal characteristics, and socioeconomic factors.

**Added value of this study**

Using nationwide health data, we emulated a target trial to estimate the real-world effectiveness of maternal RSVpreF vaccination against RSV-LRTI hospitalisation during infants’ first RSV season. Exact matching on date of birth, gestational age, sex, and deprivation status ensured strict temporal alignment between immunised and unimmunised infants. Inverse probability weighting balanced remaining measured covariates. In this population-based setting, maternal RSVpreF immunisation was associated with a 50% reduction in RSV-associated hospitalisation and an overall risk reduction for severe outcomes, including paediatric intensive care unit admission and respiratory support. Effectiveness was highest shortly after birth and declined gradually over time. Longer intervals between maternal vaccination and delivery were associated with greater effectiveness and appeared more influential than gestational timing of vaccination. The nationwide scale of the data also allowed evaluation of effectiveness across socioeconomic strata.

**Implications of all the available evidence**

Taken together with results from randomised trials and emerging real-world studies, these findings support maternal RSVpreF immunisation as an effective strategy to reduce RSV-related hospitalisation in early infancy. The observed decline in protection over time and the increased effectiveness with longer vaccination-to-delivery intervals highlights the importance of optimising vaccination timing during pregnancy to maximise infant protection during peak RSV circulation. These findings can inform policy decisions and clinical discussions on strategies to protect infants during their first RSV season, particularly in settings where long-acting monoclonal antibodies are also available for RSV prevention.

## Introduction

Respiratory syncytial virus (RSV) remains the leading cause of lower respiratory tract infection and hospitalisation in early infancy worldwide.^1^ Prevention strategies were previously limited to monoclonal antibodies administered to selected high-risk infants, leaving most newborns without protection during their first months of life.^2^ Recently, an additional preventive strategy through maternal immunisation with a bivalent prefusion F (RSVpreF) vaccine (Abrysvo®) that confers passive immunity through transplacental antibody transfer was introduced.^3^

In the phase 3 MATISSE trial, vaccination during late pregnancy reduced RSV-associated hospitalisation by 67·7% within 90 days after birth and by 56·8% at 180 days.^4^ Although randomised trials provide robust efficacy estimates, they do not fully characterise real-world effectiveness, which has thus far been assessed primarily through studies relying on test-negative or case–control designs, reporting effectiveness estimates ranging from approximately 57%–82% against RSV-associated hospitalisations.^5^, ^6^, ^7^, ^8^ While these studies extend trial findings, many were conducted in selected regions or with temporal confounding related to vaccine uptake and RSV circulation patterns. Confirmation in population-based studies, capturing the full RSV season within national immunisation programmes is therefore warranted.

The introduction of maternal vaccination has also coincided with the large-scale deployment of long-acting monoclonal antibodies for infants. During the 2024–2025 RSV season, maternal RSVpreF vaccination was implemented at national scale in France in parallel with widespread infant access to nirsevimab. While we previously reported the absolute effectiveness of nirsevimab during its first season of implementation (2023–2024 RSV season) in France^9^ and subsequently compared the relative effectiveness of maternal RSVpreF vaccination versus nirsevimab showing a lower risk of RSV-related hospitalisations with the latter (2024–2025 RSV season),^10^ neither of these analyses addressed the absolute effectiveness of maternal RSVpreF vaccination against no RSV immunisation. This question is distinct and complementary. It is essential to quantify the real-world protection conferred by maternal vaccination as a standalone strategy or when parents consider a different preventive option, and to characterise the durability of protection and the influence of vaccination timing and maternal factors on effectiveness. Using nationwide health data and a target trial emulation framework, we aimed to assess the real-world effectiveness of maternal RSVpreF vaccination against RSV-associated hospitalisation across the entire first RSV season of life in infants.

## Methods

### Data sources

Data were obtained from the French National Health Data System (SNDS), a nationwide administrative database that covers nearly the entire French population. The SNDS includes individual-level information on outpatient medical care, drug dispensations reimbursed in community pharmacies, hospital admissions, medical procedures, and vital status. Hospitalisation data were extracted from the French National Hospital Discharge Database (PMSI), which records diagnoses and procedures coded using the International Classification of Diseases, 10th Revision (ICD-10). Data from the PMSI are linked within the SNDS using pseudonymised unique individual identifiers. The nationwide coverage and longitudinal granularity of the SNDS enable large-scale pharmaco-epidemiological studies, allowing the assessment of health care utilisation and the real-world effectiveness and safety of therapeutic interventions at population scale.^11^^,^^12^

### French RSV immunisation campaign 2024–2025

During the 2024–2025 RSV immunisation campaign in France, two preventive strategies were available for the general infant population: the long-acting monoclonal antibody nirsevimab (Beyfortus®) and the maternal RSVpreF vaccine (Abrysvo®). For high-risk children younger than 2 years, palivizumab (Synagis®) remained available, although its use became very limited following the introduction of nirsevimab. The RSVpreF vaccine, fully reimbursed as of August 15, 2024, was recommended for administration between 32 and 36 weeks of gestation from September 1, 2024, to January 31, 2025. Nirsevimab was recommended for all infants during their first RSV season in outpatient settings and in maternity hospitals before discharge starting September 2024. These strategies were complementary approaches targeting different time points, during pregnancy for maternal vaccination and after birth for nirsevimab.

The decision to receive RSVpreF vaccination during pregnancy was made by parents in consultation with health-care professionals. Maternal vaccination was universally available and fully reimbursed under the national health insurance scheme, without eligibility restrictions. The national campaign concluded on January 31, 2025, following surveillance data from the French Public Health Agency indicating the end of RSV circulation.

### Study population

We identified all live-born infants recorded in the SNDS between Sept 1 and Dec 31, 2024, in mainland France, born in public hospitals to mothers aged 15–50 years at the time of delivery. Infants were eligible if they were entering their first RSV season and could be deterministically linked to their mother through national health insurance identifiers. Infants were excluded if gestational age was missing or birth information was inconsistent. Infants born to mothers who received RSVpreF during pregnancy were eligible for inclusion in the immunised group. Infants whose mothers had not received RSVpreF and had no record of RSV immunoprophylaxis during follow-up served as unimmunised comparators. Infants were excluded if their birth occurred within 14 days of maternal vaccination (accounting for 15% of all maternal RSVpreF vaccinations during the study period), as they are considered insufficiently protected and are recommended to receive monoclonal antibody administration. Infants who received nirsevimab or both strategies were excluded to isolate the effectiveness of maternal RSVpreF vaccination compared with no RSV immunoprophylaxis.

### Study design

The study was designed to emulate a pragmatic comparison between infants born to vaccinated and unvaccinated mothers.^13^ Each infant born to a mother immunised with RSVpreF was matched to one unimmunised infant on exact date of birth, gestational age (completed weeks), sex, and deprivation status defined by the French Deprivation Index (FDep).^14^ Exact matching on date of birth was prioritised to ensure alignment in calendar time, and other matching variables were selected a priori as potential confounders. Because the pool of unimmunised infants meeting the matching criteria was limited, we performed 1:1 matching with replacement.^15^ Matching with replacement allowed the same unimmunised infant to be selected at most twice, thereby preserving calendar-time alignment while maximising the number of matched pairs. This process achieved an 88% match rate (Appendix Table S1). Follow-up began at birth (time zero defined as the date of birth) and continued until the occurrence of the study outcome, death from any cause, the end of the study fixed at February 28, 2025, whichever came first. This matching strategy ensured strict alignment in calendar time and key perinatal characteristics between immunised and unimmunised infants, aligning eligibility, exposure assignment, and initiation of follow-up at birth. Hence, in accordance with established causal inference principles, eligibility and exposure assignment were defined at time zero to avoid immortal time bias.^16^

### Exposure

All community pharmacy dispensations of the RSVpreF vaccine were identified for mothers of infants born during the study period based on the specific ATC code J07BX05. RSVpreF vaccination can be prescribed by a doctor, a midwife, a pharmacist, or a nurse and administered by any of these professionals. The dispensing date was considered the date of vaccination when a pharmacist-administered vaccination was recorded; otherwise, the date of the first contact with any of the listed healthcare professionals after dispensing was considered the vaccination date. Vaccinations in the database were captured regardless of whether the prescriber and administrator were the same professional. RSVpreF vaccine uptake steadily increased throughout the study period (Appendix Figure S1).

### Outcomes

The primary outcome was hospitalisation due to RSV-associated lower respiratory tract infection (RSV-LRTI) during the infant's first RSV season. RSV-LRTI hospitalisations were identified using specific International Classification of Diseases, 10th Revision (ICD-10) codes recorded during inpatient stays, including acute RSV bronchiolitis (J210), RSV pneumonia (J121), and acute RSV bronchitis (J205). Hospitalisations were classified as RSV-related when one of these codes was recorded as the primary or related discharge diagnosis.

Other outcomes included hospitalisation for severe RSV-LRTI, defined as RSV-LRTI requiring admission to a paediatric intensive care unit (PICU) or high-dependency unit (HDU), ventilatory support (invasive or non-invasive), or supplemental oxygen during the hospital stay. Daily RSV-LRTI hospitalisation rates among all infants born in 2024 indicated that the 2024–2025 RSV season in France extended from October 2024 to February 2025, with a peak between Nov 15 and Jan 5 (Appendix Figure S1).

### Covariates

Baseline covariates measured prior to or at time zero (date of birth) included infant, maternal, and socioeconomic determinants. Infant characteristics comprised sex, gestational age, birth weight, month of birth, congenital anomalies, neonatal complications, and clinical markers associated with increased vulnerability to severe RSV infection.^17^ Maternal characteristics included age, parity, pre-existing and gestational comorbidities, proxies for smoking and alcohol status, and uptake of other recommended maternal vaccinations during pregnancy. Socioeconomic factors included area-level deprivation and indicators of health-care access, such as complementary solidarity health insurance status, consultations in maternal and child welfare centres, the French Deprivation Index (FDep)^18^ and the Localised Potential Accessibility (LPA) to general practitioners.^19^ Appendix Table S2 provides definitions for all the variables used in the study.

### Statistical analysis

After exact matching of immunised infants to unimmunised infants on date of birth, gestational age, sex, and deprivation status, we further addressed the potential bias arising from non-random assignment of maternal vaccination by estimating a propensity score using logistic regression including all prespecified baseline covariates (see parameter estimates in Appendix Table S3).^20^ Inverse probability of treatment weighting (IPTW) was applied to the matched cohort to create a weighted pseudo-population in which measured baseline characteristics were balanced between exposure groups. Covariate balance was assessed using standardised mean differences before and after matching, and after inverse probability weighting (Appendix Figure S2).^21^ Associations between maternal RSVpreF vaccination and RSV-LRTI hospitalisation were estimated using weighted Cox proportional hazards models. Hazard ratios (HRs) and corresponding 95% confidence intervals (CIs) were reported. Adjusted cumulative incidence curves were derived from the weighted Cox models and smoothed using splines with 5 knots. Vaccine effectiveness was calculated as (1-HR) × 100 with confidence interval calculated as (1-HR_Upper_) × 100 and (1-HR_Lower_) × 100, respectively.

Because matching was performed with replacement, variance estimation accounted for the induced dependence from reuse of unimmunised infants. Standard errors were estimated using cluster-robust sandwich variance estimators, with clustering at the individual level.^20^^,^^22^^,^^23^

Given evidence of non-proportional hazards, the overall HR represents a time-averaged estimate of effectiveness. To evaluate the duration of effectiveness over time since birth, we conducted predefined analyses using both interval-specific estimates (0–14, 15–30, 31–45, 46–60, 61–75, 76–90 and > 90 days) and cumulative analyses from birth, explicitly capturing the time-dependent nature of protection. Other predefined subgroup analyses were conducted according to infant sex, gestational age at birth (preterm <37 weeks vs term ≥37 weeks), socioeconomic deprivation (FDep grouped as quintiles 1–2 vs 3–5), gestational age at vaccination (<32 weeks, 32–36 weeks, and >36 weeks), interval between maternal vaccination and delivery (14–29, 30–44, 45–59, and ≥60 days), and intensity of RSV circulation (lower vs higher circulation periods defined from national surveillance data). For subgroup variables not used in matching, analyses were restricted to concordant matched pairs to preserve the matched design. Subgroup analyses were conducted within each stratum using the same analytical approach as the main analysis. Sensitivity analyses included trimming of extreme propensity score weights, multivariable regression adjustment without weighting, and restricting each unimmunised infant to a single match to evaluate the impact of matching with replacement on effectiveness estimates.

All analyses were conducted within the matched cohort, two-sided 95% confidence intervals were reported, and the study was reported in accordance with STROBE guidelines. Analyses were conducted using SAS Enterprise Guide version 9.4 (SAS Institute Inc.) and R software version 4.0.2.

### Ethics approval

According to French regulations (Public Health Code Articles L.1461–3 and R.1461–11), research using pseudonymized data from the French National Health Data System (SNDS) does not require ethics committee review or individual informed consent. Epidemiology of Health Products (EPI-PHARE) has permanent regulatory access to the SNDS, with data fully pseudonymized before access. This study was approved by the scientific committee of EPI-PHARE and registered on the EPI-PHARE study register (reference T-2025-08-637).

### Role of the funding source

No funding.

## Results

**Fig. 1:**
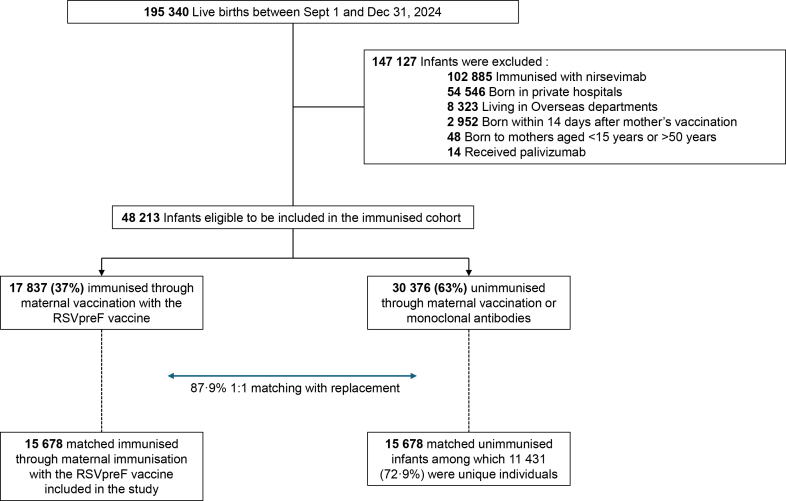
**Selection and matching of infants born to RSVpreF-vaccinated and unimmunised mothers, France, 2024–2025**.

Between September 1 and December 31, 2024, 195,340 live births were recorded in mainland France in the SNDS database. After exclusion of 147,127 infants who were immunised with nirsevimab or palivizumab, born in private hospitals or within 14 days of maternal vaccination, 48,213 infants were eligible for inclusion in the cohort. Among them, 17,837 (37%) were born to mothers vaccinated during pregnancy with RSVpreF and 30,376 (63%) were unimmunised. After 1:1 exact matching, 15,678 (87·9%) were successfully matched to unimmunised infants (Fig. 1). Among matched unimmunised infants, 11,431 (72·9%) were unique individuals; the remainder were selected more than once as controls.

A total of 31,356 infants were then included in the matched analysis (15,678 per group) with a median follow-up of 86 days (interquartile range [IQR] 73–101). By design, matched groups were identical with respect to sex, gestational age, and deprivation category. Before matching, infants in the RSVpreF group were more frequently born during periods of high RSV circulation (79·5% vs 30·4%), to primiparous mothers (52·7% vs 42·9%) and resided in less deprived areas (42·3% vs 32·6%). A larger proportion of immunised infants were born later in the RSV season. They were also less likely to benefit from complementary solidarity health insurance (10·0% vs 25·0%) and maternal and child protection services (4·9% vs 8·1%), and their mothers were substantially more likely to have received other recommended vaccines during pregnancy such as the Tdap vaccine (96·6% vs 55·9%). Indicators of neonatal vulnerability, including serious infections during the birth stay and congenital anomalies, were similar between groups (Table 1; Appendix Table S4). After matching and inverse probability weighting, infant, maternal, and socioeconomic characteristics were well balanced between groups (Appendix Figure S2).

**Table 1:** Infants and maternal characteristics at inclusion.

Characteristics	Unmatched			SMD	Matched			SMD
	Unimmunised group	RSVpreF vaccine	Total		Unimmunised group	RSVpreF vaccine	Total	
	(N = 30,376)	(N = 17,837)	(N = 48,213)		(N = 15,678)	(N = 15,678)	(N = 31,356)	
Infant characteristics								
Sex^j^—no. (%)				−0·01				<0·0001
Male	15,496 (51·0)	9208 (51·6)	24,704 (51·2)		8073 (51·5)	8073 (51·5)	16,146 (51·5)	
Female	14,880 (49·0)	8629 (48·4)	23,509 (48·8)		7605 (48·5)	7605 (48·5)	15,210 (48·5)	
Gestational age at birth^j^—no. (%)				0·20				<0·0001
Very preterm birth (<32 wk)	341 (1·1)	0 (0·0)	341 (0·7)		0 (0·0)	0 (0·0)	0 (0·0)	
Preterm birth ( ≥ 32 to <37 wk)	1320 (4·3)	325 (1·8)	1645 (3·4)		159 (1·0)	159 (1·0)	318 (1·0)	
Term birth ( ≥ 37 wk)	28,715 (94·5)	17,512 (98·2)	46,227 (95·9)		15,519 (99·0)	15,519 (99·0)	31,038 (99·0)	
Age at discharge date—d				0·05				0·11
Mean	3·6 (1·7)	3·7 (1·4)	3·7 (1·6)		3·5 (1·5)	3·7 (1·4)	3·6 (1·4)	
Median (range)	3 [3–4]	3 [3–4]	3 [3–4]		3 [3–4]	3 [3–4]	3 [3–4]	
Month of birth^j^—no. (%)				1·36				<0·0001
September	9647 (31·8)	11 (0·1)	9658 (20·0)		10 (0·1)	10 (0·1)	20 (0·1)	
October	7780 (25·6)	1266 (7·1)	9046 (18·8)		1229 (7·8)	1229 (7·8)	2458 (7·8)	
November	7094 (23·4)	6538 (36·7)	13,632 (28·3)		6084 (38·8)	6084 (38·8)	12,168 (38·8)	
December	5855 (19·3)	10,022 (56·2)	15,877 (32·9)		8355 (53·3)	8355 (53·3)	16,710 (53·3)	
Period of inclusion^j^—no. (%)				1·41				<0·0001
01–15 Sep.	5872 (19·3)	0 (0·0)	5872 (12·2)		0 (0·0)	0 (0·0)	0 (0·0)	
16–30 Sep.	3775 (12·4)	11 (0·1)	3786 (7·9)		10 (0·1)	10 (0·1)	20 (0·1)	
01–15 Oct.	3869 (12·7)	193 (1·1)	4062 (8·4)		187 (1·2)	187 (1·2)	374 (1·2)	
16–30 Oct.	3911 (12·9)	1073 (6·0)	4984 (10·3)		1042 (6·6)	1042 (6·6)	2084 (6·6)	
01–15 Nov.	3699 (12·2)	2383 (13·4)	6082 (12·6)		2276 (14·5)	2276 (14·5)	4552 (14·5)	
16–30 Nov.	3395 (11·2)	4155 (23·3)	7550 (15·7)		3808 (24·3)	3808 (24·3)	7616 (24·3)	
01–15 Dec.	3172 (10·4)	5177 (29·0)	8349 (17·3)		4399 (28·1)	4399 (28·1)	8798 (28·1)	
16–31 Dec.	2683 (8·8)	4845 (27·2)	7528 (15·6)		3956 (25·2)	3956 (25·2)	7912 (25·2)	
Birth weight^a^								
Mean (SD)—g	3276·4 (538)	3313·5 (451·8)	3290·1 (508·1)	0·08	3333·7 (446·4)	3323·5 (442·3)	3328·6 (444·4)	−0·02
Distribution—no. (%)								
Small (<P10)	3603 (11·9)	1985 (11·1)	5588 (11·6)	0·03	1590 (10·1)	1712 (10·9)	3302 (10·5)	0·04
Appropriate (P10–P90)	23,375 (77·0)	13,931 (78·1)	37,306 (77·4)		12,262 (78·2)	12,285 (78·4)	24,547 (78·3)	
Large (>P90)	3396 (11·2)	1919 (10·8)	5315 (11·0)		1824 (11·6)	1679 (10·7)	3503 (11·2)	
Region of residence—no. (%)								
Auvergne-Rhône-Alpes	3342 (11·0)	2602 (14·6)	5944 (12·3)	0·41	1801 (11·5)	2249 (14·3)	4050 (12·9)	0·53
Bourgogne-Franche-Comté	1653 (5·4)	634 (3·6)	2287 (4·7)		824 (5·3)	569 (3·6)	1393 (4·4)	
Bretagne	1235 (4·1)	1403 (7·9)	2638 (5·5)		418 (2·7)	1264 (8·1)	1682 (5·4)	
Centre-Val de Loire	1141 (3·8)	751 (4·2)	1892 (3·9)		624 (4·0)	690 (4·4)	1314 (4·2)	
Corse	153 (0·5)	53 (0·3)	206 (0·4)		75 (0·5)	49 (0·3)	124 (0·4)	
Grand Est	2472 (8·1)	1213 (6·8)	3685 (7·6)		1282 (8·2)	1097 (7·0)	2379 (7·6)	
Hauts-de-France	2924 (9·6)	2001 (11·2)	4925 (10·2)		1266 (8·1)	1801 (11·5)	3067 (9·8)	
Ile-de-France	9077 (29·9)	3231 (18·1)	12,308 (25·5)		5155 (32·9)	2728 (17·4)	7883 (25·1)	
Normandie	966 (3·2)	1132 (6·3)	2098 (4·4)		444 (2·8)	1016 (6·5)	1460 (4·7)	
Nouvelle-Aquitaine	1755 (5·8)	1460 (8·2)	3215 (6·7)		811 (5·2)	1289 (8·2)	2100 (6·7)	
Occitanie	1937 (6·4)	1298 (7·3)	3235 (6·7)		872 (5·6)	1136 (7·2)	2008 (6·4)	
Pays de la Loire	1128 (3·7)	1065 (6·0)	2193 (4·5)		539 (3·4)	909 (5·8)	1448 (4·6)	
Provence-Alpes-Côte d’Azur	2593 (8·5)	994 (5·6)	3587 (7·4)		1567 (10·0)	881 (5·6)	2448 (7·8)	
French Deprivation index^b^^,^^j^—Quintiles								
Q1–Q2 (least deprived)	9894 (32·6)	7539 (42·3)	17,433 (36·2)	0·20	6105 (38·9)	6105 (38·9)	12,210 (38·9)	<0·0001
Q3–Q4–Q5 (most deprived)	20,327 (66·9)	10,198 (57·2)	30,525 (63·3)		9561 (61·0)	9561 (61·0)	19,122 (61·0)	
Missing	155 (0·5)	100 (0·6)	255 (0·5)		12 (0·1)	12 (0·1)	24 (0·1)	
European Deprivation index^c^—Quintiles				0·41				0·36
Q1 (least deprived)	3579 (11·8)	3770 (21·3)	7349 (15·3)		1911 (12·2)	3175 (20·3)	5086 (16·2)	
Q2 (slightly deprived)	4499 (14·9)	3565 (20·1)	8064 (16·8)		2393 (15·3)	3130 (20·0)	5523 (17·6)	
Q3 (moderately deprived)	5138 (17·0)	3390 (19·1)	8528 (17·8)		2694 (17·2)	3058 (19·5)	5752 (18·4)	
Q4 (highly deprived)	6661 (22·0)	3611 (20·4)	10,272 (21·4)		3403 (21·7)	3200 (20·4)	6603 (21·1)	
Q5 (most deprived)	10,344 (34·2)	3399 (19·2)	13,743 (28·7)		5265 (33·6)	3101 (19·8)	8366 (26·7)	
General Practitioners' Localised Potential^d^—Quartiles				0·17				0·19
1 ( ≤ 3·0)	8359 (27·7)	4195 (23·6)	12,554 (26·2)		4499 (28·7)	3690 (23·6)	8189 (26·1)	
2 (3·0–3·8)	8755 (29·0)	4347 (24·5)	13,102 (27·3)		4555 (29·1)	3870 (24·7)	8425 (26·9)	
3 (3·8–4·7)	7331 (24·3)	5013 (28·3)	12,344 (25·7)		3708 (23·7)	4433 (28·3)	8141 (26·0)	
4 (>4·7)	5776 (19·1)	4183 (23·6)	9959 (20·8)		2904 (18·5)	3673 (23·4)	6577 (21·0)	
Complementary solidarity health insurance status^e^—no. (%)	7607 (25·0)	1778 (10·0)	9385 (19·5)	−0·40	3957 (25·2)	1599 (10·2)	5556 (17·7)	−0·40
Maternal and Child Protection Centres^f^—no. (%)	2473 (8·1)	878 (4·9)	3351 (7·0)	−0·13	1117 (7·1)	772 (4·9)	1889 (6·0)	−0·09
Social security affiliation type^g^—no. (%)								
General health scheme	29,305 (96·5)	17,063 (95·7)	46,368 (96·2)	0·04	15,175 (96·8)	14,993 (95·6)	30,168 (96·2)	0·06
Agricultural scheme	752 (2·5)	529 (3·0)	1281 (2·7)		340 (2·2)	477 (3·0)	817 (2·6)	
Other	319 (1·1)	245 (1·4)	564 (1·2)		163 (1·0)	208 (1·3)	371 (1·2)	
Serious infections during the birth stay^h^—no. (%)	540 (1·8)	152 (0·9)	692 (1·4)	−0·08	194 (1·2)	139 (0·9)	333 (1·1)	−0·03
Bacterial infections	505 (1·7)	136 (0·8)	641 (1·3)	−0·08	180 (1·1)	125 (0·8)	305 (1·0)	−0·04
Viral infections	32 (0·1)	17 (0·1)	49 (0·1)	−0·004	13 (0·1)	15 (0·1)	28 (0·1)	0·004
Fungal infections	21 (0·1)	4 (0·0)	25 (0·1)	−0·02	9 (0·1)	4 (0·0)	13 (0·0)	−0·02
Congenital anomalies^i^—no. (%)	444 (1·5)	185 (1·0)	629 (1·3)	−0·04	146 (0·9)	161 (1·0)	307 (1·0)	0·01
Chronic comorbidities—no. (%)								
Bronchopulmonary dysplasia (BPD)	116 (0·4)	0 (0·0)	116 (0·2)	−0·09	2 (0·0)	0 (0·0)	2 (0·0)	−0·02
Congenital heart disease (CHD)	72 (0·2)	27 (0·2)	99 (0·2)	−0·02	25 (0·2)	26 (0·2)	51 (0·2)	0·002
Maternal characteristics								
Maternal age at childbirth—years								
Mean	30·7 (5·6)	31·5 (5·1)	31 (5·4)	0·15	30·8 (5·6)	31·4 (5·1)	31·1 (5·4)	0·12
Median [IQR]	31 [27–35]	32 [28–35]	31 [27–35]		31 [27–35]	32 [28–35]	31 [27–35]	
15–24	4317 (14·2)	1607 (9·0)	5924 (12·3)	0·19	2164 (13·8)	1430 (9·1)	3594 (11·5)	0·17
25–29	8538 (28·1)	4574 (25·6)	13,112 (27·2)		4372 (27·9)	4066 (25·9)	8438 (26·9)	
30–34	9735 (32·0)	6587 (36·9)	16,322 (33·9)		5032 (32·1)	5788 (36·9)	10,820 (34·5)	
35–49	7786 (25·6)	5069 (28·4)	12,855 (26·7)		4110 (26·2)	4394 (28·0)	8504 (27·1)	
Maternal Parity								
1	13,017 (42·9)	9403 (52·7)	22,420 (46·5)	0·32	6571 (41·9)	8246 (52·6)	14,817 (47·3)	0·32
2	10,140 (33·4)	6165 (34·6)	16,305 (33·8)		5334 (34·0)	5440 (34·7)	10,774 (34·4)	
3	4578 (15·1)	1706 (9·6)	6284 (13·0)		2443 (15·6)	1484 (9·5)	3927 (12·5)	
≥4	2641 (8·7)	563 (3·2)	3204 (6·6)		1330 (8·5)	508 (3·2)	1838 (5·9)	
Mode of delivery				0·004				0·02
Vaginal	24,083 (79·8)	14,135 (79·6)	38,218 (79·8)		12,575 (80·8)	12,470 (79·9)	25,045 (80·3)	
Caesarean	6087 (20·2)	3612 (20·4)	9699 (20·2)		2994 (19·2)	3131 (20·1)	6125 (19·7)	
Length of birth hospitalisation—d.								
Mean	4·5 (2·8)	4·5 (1·9)	4·5 (2·5)	−0·02	4·3 (2)	4·4 (1·8)	4·4 (1·9)	0·07
Median (range)	4 [3–5]	4 [3–5]	4 [3–5]		4 [3–5]	4 [3–5]	4 [3–5]	
Time between vaccination and childbirth—w.								
Mean	–	5·2 (1·8)	–		–	5·2 (1·8)	–	
Median (IQR)	–	5·0 (3·9–6·1)	–		–	5·0 (3·9–6·1)	–	
Other maternal vaccines								
TDAP	16,979 (55·9)	17,229 (96·6)	34,208 (71·0)	1·09	8484 (54·1)	15,161 (96·7)	23,645 (75·4)	1·14
Influenza vaccine	544 (1·8)	4962 (27·8)	5506 (11·4)	0·79	521 (3·3)	4196 (26·8)	4717 (15·0)	0·69
SARS-CoV-2 vaccine	204 (0·7)	1578 (8·8)	1782 (3·7)	0·39	139 (0·9)	1347 (8·6)	1486 (4·7)	0·37
Maternal comorbidities								
Pre-existing diabetes	271 (0·9)	157 (0·9)	428 (0·9)	−0·001	138 (0·9)	135 (0·9)	273 (0·9)	−0·002
Gestational diabetes	5037 (16·6)	2655 (14·9)	7692 (16·0)	−0·05	2495 (15·9)	2351 (15·0)	4846 (15·5)	−0·03
Chronic hypertension	416 (1·4)	310 (1·7)	726 (1·5)	0·03	206 (1·3)	273 (1·7)	479 (1·5)	0·03
Pre-eclampsia	1266 (4·2)	688 (3·9)	1954 (4·1)	−0·02	573 (3·7)	592 (3·8)	1165 (3·7)	0·006
Obesity	3174 (10·4)	1653 (9·3)	4827 (10·0)	−0·04	1593 (10·2)	1483 (9·5)	3076 (9·8)	−0·02
Lifestyle habits								
Tobacco use	3481 (11·5)	2109 (11·8)	5590 (11·6)	0·01	1623 (10·4)	1835 (11·7)	3458 (11·0)	0·04
Alcohol consumption	179 (0·6)	100 (0·6)	279 (0·6)	−0·004	87 (0·6)	76 (0·5)	163 (0·5)	−0·01
Opiates intake	61 (0·2)	24 (0·1)	85 (0·2)	−0·02	29 (0·2)	22 (0·1)	51 (0·2)	−0·01
RSV activity level at time of birth—no. (%)								
High	9246 (30·4)	14,173 (79·5)	23,419 (48·6)	1·13	12,162 (77·6)	12,162 (77·6)	24,324 (77·6)	<0·0001
Low	21,130 (69·6)	3664 (20·5)	24,794 (51·4)		3516 (22·4)	3516 (22·4)	7032 (22·4)	

Abbreviations: RSV = respiratory syncytial virus; RSVpreF = respiratory syncytial virus prefusion F protein vaccine; SMD = standardised mean difference, values <0·1 indicate negligible imbalance; TDAP = tetanus, diphtheria, and acellular pertussis vaccine; GPLP = General Practitioners' Localised Potential; wk = weeks; Q1–Q3 = first and third quartiles.

a Birth weight percentiles: Defined according to national reference growth charts for gestational age; small for gestational age (SGA) corresponds to <P10, appropriate for gestational age (AGA) to P10–P90, and large for gestational age (LGA) to >P90.

b The French Deprivation Index: an ecological, area-based measure of social disadvantage in France, constructed at the commune level using four standardised variables (median household income per consumption unit, percentage of employed population in manual occupations, unemployment rate, and percentage of adults without a baccalaureate). Higher quintiles values indicating greater deprivation.

c European Deprivation index: An ecological, small-area indicator of relative socioeconomic deprivation developed using harmonised methods across European countries. Higher EDI values indicate greater deprivation, categorised into quintiles from Q1 (least deprived) to Q5 (most deprived).

d General Practitioners' Localised Potential: Indicator of access to primary care physicians, calculated from the density of general practitioners relative to population needs in each area; higher quartiles indicate greater access.

e Complementary solidarity health insurance status: National public insurance program providing full coverage of health care costs for low-income individuals and families.

f Maternal and Child Protection Centres: Public community health services providing preventive medical care and social support to mothers and infants.

g Social security affiliation type: Categorised according to the national insurance scheme: general, agricultural, or other special schemes.

h Serious infections during the birth stay: Includes bacterial, viral, or fungal infections documented during the initial hospitalisation following delivery identified using International Classification of Diseases, Tenth Revision (ICD-10) details are provided in Appendix Table S2.

i Congenital anomalies: Major structural or chromosomal anomalies. The full list of ICD-10 (CIM-10) codes used for classification is provided in Appendix Table S2, and the frequency and description of individual anomalies are detailed in Appendix Table S4. Major anomaly categories include: congenital heart defects and diseases, nervous system and chromosomal abnormalities, respiratory and oesophageal abnormalities, digestive system and abdominal wall defects, renal urinary and genital malformations, limb and musculoskeletal anomalies, craniofacial anomalies, other teratogenic anomalies.

j Indicates variables used in the matching process.

A total of 693 RSV-LRTI hospitalisations were recorded during the study period. Among them, 477 (3·0%) occurred in unimmunised infants and 216 (1·4%) in infants born to mothers vaccinated with RSVpreF (p < 0·001). Bronchiolitis accounted for most hospitalisations in both groups (99·2% vs 96·8%). Median length of stay was shorter among immunised infants (5 [4–8] vs 6 [4–9]; p < 0·0005), with fewer prolonged hospitalisations (>1 week; 27·8% vs 36·3%). Once hospitalised for RSV-LRTI, admission to a paediatric intensive care unit (34·3% vs 33·1%; p = 0·77), oxygen therapy (27·3% vs 28·5%; p = 0·71), and ventilatory support (30·1% vs 28·7%; p = 0·75), were comparable between groups. No in-hospital deaths were recorded (Table 2).

**Table 2:** Secondary Outcomes and characteristics of hospitalisations for Respiratory Syncytial Virus-Associated Lower Respiratory Tract Infection (RSV- LRTI).

Hospitalisation characteristics	Hospitalisation for RSV-LRTI			Difference per 100	p-value
	Unimmunised group	RSVpreF vaccine	All		
	(N = 477) 68·8%	(N = 216) 31·2%	(N = 693) 100%	95% (CI)	
RSV-associated lower respiratory tract infection—no. (%)					
Bronchiolitis	473 (99·2)	209 (96·8)	682 (98·4)	−2·4 [−4·9 to 0·1]	0·019
Bronchitis	4 (0·8)	7 (3·2)	11 (1·6)	2·4 [−0·1 to 4·9]	
Pneumonitis	0 (0·0)	0 (0·0)	0 (0·0)	0·0 (0·0–0·0)	
Length of inpatient hospitalisation					
Mean (SD)—d.	8·3 (9·2)	6·8 (6·6)	7·8 (8·5)		0·033
Median [IQR]—d.	6 [4–9]	5 [4–8]	6 [4–9]		0·0005
Distribution—no. (%)					
1 day to 1 wk	304 (63·7)	156 (72·2)	460 (66·4)	8·5 [1·1–15·9]	0·028
>1 wk	173 (36·3)	60 (27·8)	233 (33·6)	−8·5 [−15·9 to −1·1]	
PICU and/or HDU admission—no. (%)	263 (55·1)	113 (52·3)	376 (54·3)	−2·8 [−10·8 to 5·2]	0·49
Paediatric Intensive Care Unit admission—no. (%)	158 (33·1)	74 (34·3)	232 (33·5)	1·1 [−6·5 to 8·7]	0·77
Paediatric High Dependency Unit^a^—no. (%)	143 (30·0)	50 (23·1)	193 (27·8)	−6·8 [−13·8 to 0·1]	0·063
Ventilatory support—no. (%)	137 (28·7)	65 (30·1)	202 (29·1)	1·4 [−6·0 to 8·7]	0·71
Non-invasive ventilation	123 (25·8)	63 (29·2)	186 (26·8)	3·4 [−3·8 to 10·6]	0·35
Invasive ventilation	14 (2·9)	2 (0·9)	16 (2·3)	−2 [−4 to 0]	0·17
Oxygen therapy—no. (%)	136 (28·5)	59 (27·3)	195 (28·1)	−1·2 [−8·4 to 6·0]	0·75
Extracorporeal Membrane Oxygenation (ECMO)—no. (%)	0 (0·0)	0 (0·0)	0 (0·0)	0·0 (0·0–0·0)	–
Administration of nitric oxide—no. (%)	2 (0·4)	0 (0·0)	2 (0·3)	−0·4 [−1 to 0·2]	0·85
Hospital death—no. (%)	0 (0·0)	0 (0·0)	0 (0·0)	0·0 (0·0–0·0)	–

Abbreviations: RSV, respiratory syncytial virus; RSV-LRTI, respiratory syncytial virus-associated lower respiratory tract infection; PICU, paediatric intensive care unit; HDU, high-dependency unit; SD, standard deviation; Q1–Q3, first and third quartiles.

a A high-dependency unit (HDU) refers to a hospital care area providing continuous monitoring and specialised care for infants requiring a higher level of observation and treatment than standard paediatric wards but not full intensive care.

**Fig. 2:**
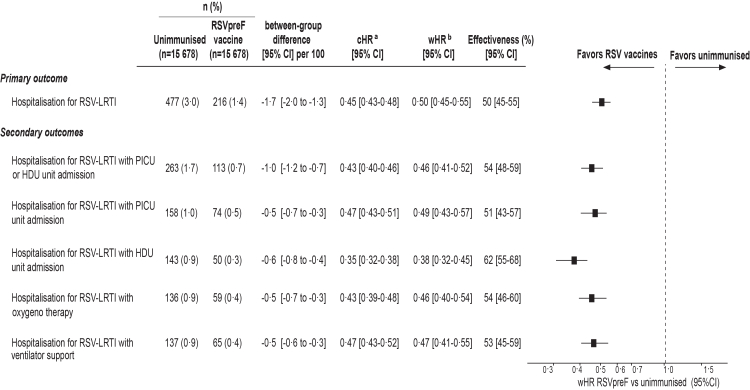
**Effectiveness of the maternal RSVpreF vaccination against RSV-associated hospitalisation among infants.** Abbreviations: RSV-LRTI, respiratory syncytial virus-associated lower respiratory tract infection; PICU, paediatric intensive care unit; HDU, high-dependency unit (hospital care area providing continuous monitoring and specialised care for infants requiring a higher level of observation and treatment than standard paediatric wards but not full intensive care). a. cHR: Crude hazard ratio corresponds to the matched cohort before weighting. Matching was performed on four variables date of birth, sex, gestational age, and deprivation index. b. wHR: Weighted hazard ratio derived using an inverse probability of treatment weighting (IPTW) method applied to the matched dataset to further balance residual covariates.

Maternal vaccination was associated with a lower risk of RSV-LRTI hospitalisation (weighted HR 0·50, 95% CI 0·45–0·55), corresponding to an effectiveness of 50% (95% CI 45–55) and an absolute risk reduction of 1·7 cases per 100 individuals. Reductions were also observed for severe outcomes, including RSV-LRTI requiring paediatric intensive care unit admission alone (51%, 95% CI 43–57), and high-dependency unit admission (62%, 95% CI 55–68). RSV-LRTI requiring oxygen therapy (54%, 95% CI 46–60) and ventilatory support (53%, 95% CI 45–59) were likewise associated with reduced risk (Fig. 2; Appendix Figure S3).

**Fig. 3:**
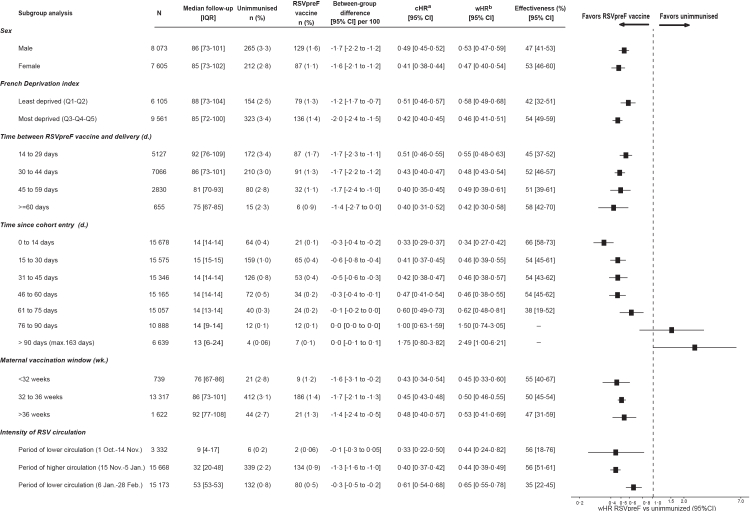
**Subgroup analyses of the effectiveness of the maternal RSVpreF vaccination against RSV-associated LRTI hospitalisations among infants.** Abbreviations: RSV-LRTI, respiratory syncytial virus-associated lower respiratory tract infection; HR, hazard ratio; wHR, weighted hazard ratio; Q1,Q5, firstto fifth quintiles; FDep, French Deprivation Index. a. cHR: Crude hazard ratio corresponds to the matched cohort before weighting. Matching was performed on four variables: sex, gestational age, day of discharge from the maternity ward, and region of residence. b. wHR: Weighted hazard ratio derived using an inverse probability of treatment weighting (IPTW) method applied to the matched dataset to further balance residual covariates.

**Fig. 4:**
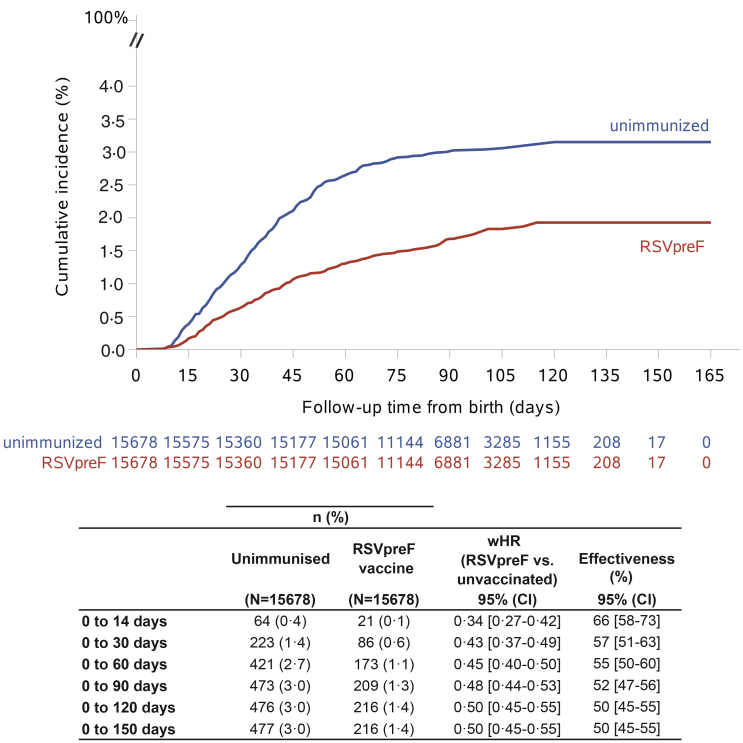
**Cumulative incidence for RSV-associated hospitalisation among infants.** wHR: Weighted hazard ratio; derived using an inverse probability of treatment weighting (IPTW) method applied to the matched dataset to further balance residual covariates.

Analyses by time since birth showed higher effectiveness in the earliest period of life, followed by a progressive decline over time. During days 0–14, effectiveness was 66% (95% CI 58–73), decreasing to 54% and remaining stable during days 15–30 (95% CI 45–61), 31–45 (95% CI 43–62), and 46–60 (95% CI 45–62). The effectiveness further decreased thereafter to 38% (95% CI 19–52) during days 61–75, and beyond 75 days of life, estimates no longer suggested protection (Fig. 3). Cumulative analyses from birth showed similarly a progressive attenuation of effectiveness over time, decreasing from 66% at 14 days to 57% (95% CI 51–63) at 30 days, 55% (95% CI 50–60) at 60 days, and 52% (95% CI 47–56) at 90 days, stabilising at 50% (95% CI 45–55) by 120–150 days (Fig. 4).

Effectiveness was broadly consistent across predefined subgroups (Fig. 3). It was observed in both male (47%, 95% CI 41–53) and female infants (53%, 95% CI 46–60). Among infants born at term, effectiveness was 51% (95% CI 46–56), whereas estimates in preterm infants were imprecise owing to small numbers. Effectiveness appeared greater among infants residing in the most deprived areas (54%, 95% CI 49–59) than among those in the least deprived areas (42%, 95% CI 32–51). When stratified by interval between maternal vaccination and delivery, effectiveness appeared to increase with longer vaccination-to-birth intervals: 45% for 14–29 days (95% CI 37–52), 52% for 30–44 days (95% CI 46–57), 51% for 45–59 days (95% CI 39–61), and 58% for ≥60 days (95% CI 42–70). Across gestational timing windows of maternal vaccination, a modest decrease of effectiveness was observed (<32 weeks: 55% (95% CI 40–67); 32–36 weeks: 50% (95% CI 45–54); >36 weeks: 47% (95% CI 31–59)), although with overlapping confidence intervals (Fig. 3).

Sensitivity analyses yielded similar results. Restricting the analysis to matched sets in which each unimmunised infant was considered only once provided similar estimates (51%, 95% CI 47–55) (Appendix Table S5).

## Discussion

In this nationwide population-based study, maternal RSVpreF immunisation was associated with a 50% reduction in RSV-associated lower respiratory tract infection hospitalisations during infants’ first RSV season compared with no RSV immunisation. Effectiveness was highest in the first weeks of life (66% at 14 days after birth) and declined progressively thereafter, with cumulative estimates decreasing to about 50% by 120 days after birth. Reductions were also observed for severe outcomes, including paediatric intensive care unit admission (51%), high-dependency unit admission (62%), ventilatory support (53%), and oxygen therapy (54%). Effectiveness also increased with longer intervals between maternal vaccination and delivery.

Our results extend previous evidence by confirming, at a national level and in real-world conditions, an association between maternal RSVpreF immunisation and reduced risk of hospitalisation for RSV-associated LRTI. Our population-level effectiveness estimates of 50% lies within, although toward the lower bound of, the range reported in the MATISSE trial^4^ and other observational studies.^5^, ^6^, ^7^, ^8^ The United Kingdom and Scotland recommended maternal vaccination from 28 weeks of gestation, earlier than the 32–36 week window implemented in France, which may have contributed to longer vaccination-to-delivery intervals and higher observed effectiveness in these settings. However, the gestational window recommended in the United States is similar to that in France, yet effectiveness estimates from US studies were also higher, suggesting that vaccination timing alone does not fully explain cross-setting differences. Such variability across settings may relate to differences in vaccine coverage, implementation timing, or population sociodemographic profiles. In contexts with high uptake of infant immunoprophylaxis, the remaining unimmunised population may have a lower underlying risk of infection due to indirect protection.^24^ Differences in study design may also contribute to variability in estimates. Test-negative and case–control approaches, although widely used may remain vulnerable to selection bias.

Maternal RSVpreF immunisation was significantly associated with shorter hospital stays and fewer prolonged hospitalisations, suggesting possible attenuation of disease severity. However, among infants who were hospitalised, the proportions requiring paediatric intensive care, ventilatory support, or oxygen therapy were similar between groups. In absolute terms, effectiveness against RSV-associated hospitalisation requiring PICU admission was 51%, reflecting the reduction in the overall risk of hospital admissions rather than a differential effect on in-hospital severity. Once hospitalised for RSV, immunised infants had a probability of PICU admission comparable to that of unimmunised infants. This pattern contrasts with our previous findings, in which infants receiving nirsevimab who were hospitalised for RSV remained at lower risk of severe in-hospital outcomes than those exposed to maternal RSVpreF vaccination,^11^ suggesting that maternal immunisation might primarily reduce the risk of hospital admission rather than substantially modifying disease severity after admission.

The attenuation of protection over time observed in our study is consistent with the MATISSE phase 3 trial, in which vaccine efficacy against RSV-associated hospitalisation was 67·7% within 90 days after birth and 56·8% within 180 days. In our analysis, interval estimates indicated that effectiveness was highest in the first weeks of life and declined thereafter, consequently cumulative analyses showed a gradual reduction in protection from 66% at 14 days to about 50% by 120–150 days of life. Although this likely reflects the natural decay of maternally derived antibodies, it has important implications for the timing of maternal vaccination.^25^ In France, the national rollout of RSVpreF began in early September 2024, at the start of the RSV season, which typically peaks between November and December. Because protection was strongest in the first weeks of life and attenuated thereafter, vaccination timing during pregnancy may be critical to ensure that infants are optimally protected during peak viral circulation. Similar seasonal recommendations have been adopted in other countries like the United States, where vaccination is advised during the RSV season (September to January).^26^

Effectiveness increased with longer vaccination-to-delivery intervals in our study. However, it remained of similar magnitude across gestational timing windows, despite slightly higher estimates for vaccination administered before 32 weeks (55%) and a marginal decrease in effectiveness after 36 weeks of gestation (47%). Longer vaccination-to-birth intervals allow for optimal transplacental transfer of RSV-specific antibodies, resulting in higher cord blood antibody concentrations at birth. Previous studies showed that antibody transfer is driven primarily by the vaccination-to-delivery interval rather than gestational age at immunisation, with optimal transfer observed when vaccination occurred at least five weeks before birth.^14^^,^^25^ In real-world settings, this interval remains constrained by the unpredictability of delivery timing, recommendations to vaccinate after 32 weeks’ gestation, and other factors like maternal IgG concentrations may also influence antibody transfer efficiency.^25^^,^^27^ Earlier vaccination in pregnancy may also extend the interval between vaccination and delivery, potentially enhancing transplacental antibody transfer.

Interestingly, effectiveness appeared greater among infants residing in more socioeconomically deprived areas. This pattern is consistent with longstanding evidence that infants from disadvantaged households are at higher risk of RSV infection and hospitalisation, as shown in early cohort studies and more recent population-based analyses of socioeconomic gradients in RSV-related admissions.^28^^,^^29^ Higher baseline risk in these settings translates into larger absolute benefits from maternal vaccination. The greater relative effectiveness observed in deprived areas should however be interpreted cautiously, as confidence intervals across deprivation strata overlapped and residual confounding cannot be excluded. In our previous studies examining uptake of RSV preventive strategies, we observed that infants living in the most deprived areas were however paradoxically less likely to receive any RSV immunisation, whether nirsevimab or maternal RSVpreF vaccination.^30^^,^^31^ Together, these findings suggest that maternal vaccination has the potential to attenuate social disparities in RSV morbidity provided that equitable uptake is achieved.

This study has several limitations. First, because maternal RSVpreF vaccination became available in September 2024 and uptake increased progressively during the campaign, infants born to vaccinated mothers were more frequently born later in the RSV season, particularly during periods of higher viral circulation. Although exact matching on date of birth ensured strict calendar-time alignment between groups, the overall cohort is shifted toward later-season births, which may limit generalisability to infants born earlier in the season. Excluded infants, mainly due to receipt of other RSV immunoprophylaxis, may further limit generalisability to the overall birth cohort. Second, follow-up was restricted to a single RSV season and to first-season outcomes. Third, estimates in preterm infants were imprecise because of small numbers. This limited precision reflects the recommended vaccination window between 32 and 36 weeks’ gestation, which inherently results in a low number of very preterm births occurring after maternal immunisation. Fourth, RSV-associated hospitalisations were identified using ICD-10 discharge codes; individual-level virological test results are not available in the database. Although RSV testing is routinely performed in hospitalised infants during the RSV season in France, limited sensitivity may have led to under-ascertainment of cases coded under non-specific respiratory diagnoses. Fifth, despite matching and inverse probability weighting to balance measured covariates, residual confounding and selection bias, particularly related to unmeasured health-seeking behaviours, language, ethnicity, or vaccine acceptance, could not be entirely excluded. Finally, these findings reflect the effectiveness of maternal RSVpreF vaccination as implemented in routine practice within a national immunisation programme and should be interpreted in that context. The concurrent implementation of nirsevimab may have influenced RSV circulation and infection risk. However, evidence for herd immunity effects remains limited, and substantial interruption of transmission is unlikely.

This nationwide study used the French National Health Data System, capturing almost all births, maternal vaccinations, and hospitalisations at a population level enabling generalisability and limiting selection biases. The target trial emulation design, aligning eligibility, exposure assignment, and follow-up at birth minimised immortal time bias. Exact matching on perinatal and socioeconomic variables ensured comparability, and inverse probability weighting balanced the remaining measured covariates. Effectiveness was assessed across a full RSV season within a national immunisation programme, reflecting real-world utilisation. Another strength of this study is the detailed hospital data which allowed evaluation of indicators of severity within the RSV-LRTI hospital stays, including intensive care admission and respiratory support. Finally, a key strength of this study is the inclusion of subgroup analyses, allowing assessment of heterogeneity in effectiveness across clinically relevant subgroups, including social deprivation, gestational timing of maternal vaccination, vaccination-to-delivery interval, and durability of protection after birth. These estimates help provide answers to clinicians and parents when choosing the optimal timing and best RSV immunisation strategy for the infant.

### Conclusion

These findings provide the first large-scale real-world estimate of the absolute effectiveness of RSVpreF in a universal health-care system. Maternal RSVpreF immunisation was associated with a reduction in RSV-associated hospitalisations during the first RSV season of life. Protection was strongest in the earliest weeks after birth and attenuated over time. These findings have important implications for RSV prevention policy. In a context where both maternal vaccination and infant monoclonal antibodies are available, understanding durability and timing of protection is essential to optimise implementation strategies.

## Contributors

MJJ, MB, AG, LT, EK, AR, VO, and MZ (all authors) conceived and designed the experiments. MJJ and MB analysed the data. MJJ and MB interpreted the results. MJJ and MB wrote the first and the revised drafts of the manuscript. All the authors contributed to the writing of the manuscript. All the authors agreed with the results and conclusions of the manuscript. All authors have read, and confirm that they meet, ICMJE criteria for authorship. MJJ and MB had full access to raw data. All authors had full access to all of the data (including statistical reports and tables) in the study and can take responsibility for the integrity of the data and the accuracy of the data analysis. MZ is the guarantor.

## Data sharing statement

According to data protection and the French regulation, the authors cannot publicly release the data from the French national health data system (SNDS). However, any person or organisation, public or private, for-profit or non-profit, is able to access SNDS data upon authorisation from the French Data Protection Office (CNIL) to carry out a study, research, or an evaluation of public interest.

## Declaration of interests

All authors declare no competing interests.
