# Supplementary material for: Maternal RSVpreF Immunisation Against Infant RSV Hospitalisation: Nationwide Population-Based Effectiveness and Durability Study
Source: Lancet Reg Health Eur. 2026 Jun 20;67:101756. doi: 10.1016/j.lanepe.2026.101756 (PMC13310600; doi:10.1016/j.lanepe.2026.101756)
Supplement: Supplementary Material [file mmc1.docx]

**Appendix.** Effectiveness and durability of maternal RSVpreF vaccination against infant RSV hospitalisation during the first RSV season: a nationwide study in France

**Table des matières**

[**Table S1.** Baseline characteristics before and after matching among infants exposed to maternal RSVpreF vaccination 3](#_Toc223946979)

[**Table S2**. Variables’ definition 6](#_Toc223946980)

[**Table S3.** Parameter estimates from the propensity score analysis 11](#_Toc223946981)

[**Table S4.** Extensive Infants and maternal characteristics at inclusion 13](#_Toc223946982)

[**Table S5.** Sensitivity analysis for the effectiveness of the maternal RSVpreF vaccination against RSV-associated hospitalization 15](#_Toc223946983)

[**Figure S1.** Temporal trends in RSV-related infant hospitalisations in France during the 2024–2025 season among all infants born in 2024 (N = 650,000), and daily numbers of infants born to mothers immunised with RSVpreF and to unimmunised mothers from Sept 1 to Dec 31, 2024. 16](#_Toc223946984)

[**Figure S2.** Standardized mean differences before and after inverse probability of treatment weighting 17](#_Toc223946985)

[18](#_Toc223946986)

[**Figure S3.** Hospitalization for RSV-related lower respiratory tract infection (RSV-LRTI) by group using IPTW Kaplan-Meier curves 18](#_Toc223946987)

[**Appendix Methods.** Specification of the Target Trial and Its Emulation 19](#_Toc223946988)

# **Table S1.** Baseline characteristics before and after matching among infants exposed to maternal RSVpreF vaccination

|  | **RSVpreF vaccine** | |  |
| --- | --- | --- | --- |
|  | **Unmatched** | **Matched** | **Total** |
| **Characteristics** | **(N = 2159)** | **(N = 15678)** | **17837** |
|  | **12%** | **88%** | **100%** |
|  |  |  |  |
| *Infant characteristics* |  |  |  |
| **Sex — no. (%)** |  |  |  |
| Male | 1135 (52.6) | 8073 (51.5) | 9208 (51.6) |
| Female | 1024 (47.4) | 7605 (48.5) | 8629 (48.4) |
|  |  |  |  |
| **Gestational age at birth — no. (%)** |  |  |  |
| Very preterm birth (<32 wk) | 0 (0.0) | 0 (0.0) | 0 (0.0) |
| Preterm birth (≥32 to <37 wk.) | 166 (7.7) | 159 (1.0) | 325 (1.8) |
| Term birth (≥37 wk.) | 1993 (92.3) | 15519 (99.0) | 17512 (98.2) |
|  |  |  |  |
| **Month of birth — no. (%)** |  |  |  |
| September | 1 (0.0) | 10 (0.1) | 11 (0.1) |
| October | 37 (1.7) | 1229 (7.8) | 1266 (7.1) |
| November | 454 (21.0) | 6084 (38.8) | 6538 (36.7) |
| December | 1667 (77.2) | 8355 (53.3) | 10022 (56.2) |
|  |  |  |  |
| **Gestation at RSVpreF vaccine injection — wk** | 34.2 (1.9) | 34.6 (1.7) | 34.5 (1.7) |
| **Gestation at RSVpreF vaccine injection — med** | 34.4 [33.1-35.4] | 34.9 [33.6-35.7] | 34.7 [33.6-35.7] |
| Within 32–36 weeks — no. (%) | 1806 (83.6) | 13317 (84.9) | 15123 (84.8) |
|  |  |  |  |
| **Birth weight** | 3240.7 (509.4) | 3323.5 (442.3) | 3313.5 (451.8) |
| Small (<P10) | 273 (12.6) | 1712 (10.9) | 1985 (11.1) |
| Appropriate (P10-P90) | 1646 (76.2) | 12285 (78.4) | 13931 (78.1) |
| Large (>P90) | 240 (11.1) | 1679 (10.7) | 1919 (10.8) |
|  |  |  |  |
| **Region of residence — no. (%)** |  |  |  |
| Auvergne-Rhône-Alpes | 353 (16.4) | 2249 (14.3) | 2602 (14.6) |
| Bourgogne-Franche-Comté | 65 (3.0) | 569 (3.6) | 634 (3.6) |
| Bretagne | 139 (6.4) | 1264 (8.1) | 1403 (7.9) |
| Centre-Val de Loire | 61 (2.8) | 690 (4.4) | 751 (4.2) |
| Corse | 4 (0.2) | 49 (0.3) | 53 (0.3) |
| Grand Est | 116 (5.4) | 1097 (7.0) | 1213 (6.8) |
| Hauts-de-France | 200 (9.3) | 1801 (11.5) | 2001 (11.2) |
| Ile-de-France | 503 (23.3) | 2728 (17.4) | 3231 (18.1) |
| Normandy | 116 (5.4) | 1016 (6.5) | 1132 (6.3) |
| Nouvelle-Aquitaine | 171 (7.9) | 1289 (8.2) | 1460 (8.2) |
| Occitanie | 162 (7.5) | 1136 (7.2) | 1298 (7.3) |
| Pays de la Loire | 156 (7.2) | 909 (5.8) | 1065 (6.0) |
| Provence-Alpes-Côte d’Azur | 113 (5.2) | 881 (5.6) | 994 (5.6) |
|  |  |  |  |
| **French Deprivation index* — Quintiles** |  |  |  |
| Q1 - Q2 (least deprived) | 1434 (66.4) | 6105 (38.9) | 7539 (42.3) |
| Q3 - Q4 - Q5 (most deprived) | 637 (29.5) | 9561 (61.0) | 10198 (57.2) |
| Missing | 88 (4.1) | 12 (0.1) | 100 (0.6) |
|  |  |  |  |
| **European Deprivation index — Quintiles** |  |  |  |
| Q1 (least deprived) | 595 (28.7) | 3175 (20.3) | 3770 (21.3) |
| Q2 (slightly deprived) | 435 (21.0) | 3130 (20.0) | 3565 (20.1) |
| Q3 (moderately deprived) | 332 (16.0) | 3058 (19.5) | 3390 (19.1) |
| Q4 (highly deprived) | 411 (19.8) | 3200 (20.4) | 3611 (20.4) |
| Q5 (most deprived) | 298 (14.4) | 3101 (19.8) | 3399 (19.2) |
|  |  |  |  |
| **General Practitioners’ Localized Potential — Quartiles** |  |  |  |
| 1 (<=3,0) | 505 (24.4) | 3690 (23.6) | 4195 (23.6) |
| 2 (3,0-3.8) | 477 (23.0) | 3870 (24.7) | 4347 (24.5) |
| 3 (3.8-4.7) | 580 (28.0) | 4433 (28.3) | 5013 (28.3) |
| 4 (>4.7) | 510 (24.6) | 3673 (23.4) | 4183 (23.6) |
|  |  |  |  |
| **Complementary solidarity health insurance status — no. (%)** | 179 (8.3) | 1599 (10.2) | 1778 (10.0) |
|  |  |  |  |
| **Maternal and Child Protection Centers (PMI) — no. (%)** | 106 (4.9) | 772 (4.9) | 878 (4.9) |
|  |  |  |  |
| **Social security affiliation type — no. (%)** |  |  |  |
| General health scheme | 2070 (95.9) | 14993 (95.6) | 17063 (95.7) |
| Agricultural scheme | 52 (2.4) | 477 (3.0) | 529 (3.0) |
| Other | 37 (1.7) | 208 (1.3) | 245 (1.4) |
|  |  |  |  |
| **Congenital anomalies — no. (%)** | 24 (1.1) | 161 (1.0) | 185 (1.0) |
|  |  |  |  |
| **Serious infections during the birth stay — no. (%)** | 13 (0.6) | 139 (0.9) | 152 (0.9) |
|  |  |  |  |
| **Mode of delivery - no. (%)** |  |  |  |
| Vaginal | 1665 (77.6) | 12470 (79.9) | 14135 (79.6) |
| Cesarean | 481 (22.4) | 3131 (20.1) | 3612 (20.4) |
|  |  |  |  |
| **Length of birth hospitalization — d.** | 4.8 (2.6) | 4.4 (1.8) | 4.5 (1.9) |
| **Median length of birth hospitalization [IQR]** | 4 [3-5] | 4 [3-5] | 4 [3-5] |
|  |  |  |  |
| **Time between mother vaccination and childbirth — wk** | 5.2 (1.9) | 5.2 (1.7) | 5.2 (1.8) |
| **Median time between mother vaccination and childbirth [IQR]** | 5 [3.7-6.3] | 5 [3.9-6.1] | 5 [3.9-6.1] |
|  |  |  |  |
| **RSV activity level at time of birth — no. (%)** |  |  |  |
| High | 2011 (93.1) | 12162 (77.6) | 14173 (79.5) |
| Low | 148 (6.9) | 3516 (22.4) | 3664 (20.5) |
|  |  |  |  |
| *Maternal characteristics* |  |  |  |
|  |  |  |  |
| **Maternal age at childbirth — years** |  |  |  |
| Mean (SD) | 32 (5.2) | 31.4 (5.1) | 31.5 (5.1) |
| Median [IQR] | 32 [29-36] | 32 [28-35] | 32 [28-35] |
| 15-24 | 177 (8.2) | 1430 (9.1) | 1607 (9.0) |
| 25-29 | 508 (23.5) | 4066 (25.9) | 4574 (25.6) |
| 30-34 | 799 (37.0) | 5788 (36.9) | 6587 (36.9) |
| 35-49 | 675 (31.3) | 4394 (28.0) | 5069 (28.4) |
|  |  |  |  |
| **Maternal Parity - no. (%)** |  |  |  |
| 1 | 1157 (53.6) | 8246 (52.6) | 9403 (52.7) |
| 2 | 725 (33.6) | 5440 (34.7) | 6165 (34.6) |
| 3 | 222 (10.3) | 1484 (9.5) | 1706 (9.6) |
| ≥ 4 | 55 (2.5) | 508 (3.2) | 563 (3.2) |
|  |  |  |  |
| **Other maternal vaccines - no. (%)** |  |  |  |
| TDAP | 2068 (95.8) | 15161 (96.7) | 17229 (96.6) |
| Influenza vaccine | 766 (35.5) | 4196 (26.8) | 4962 (27.8) |
| SARS-CoV-2 vaccine | 231 (10.7) | 1347 (8.6) | 1578 (8.8) |
|  |  |  |  |
| **Maternal comorbidities- no. (%)** |  |  |  |
| Pre-existing diabetes | 22 (1.0) | 135 (0.9) | 157 (0.9) |
| Gestational diabetes | 304 (14.1) | 2351 (15.0) | 2655 (14.9) |
| Chronic hypertension | 37 (1.7) | 273 (1.7) | 310 (1.7) |
| Pre-eclampsia | 96 (4.4) | 592 (3.8) | 688 (3.9) |
| Obesity | 170 (7.9) | 1483 (9.5) | 1653 (9.3) |
|  |  |  |  |
| **Lifestyle habits- no. (%)** |  |  |  |
| Tobacco use | 274 (12.7) | 1835 (11.7) | 2109 (11.8) |
| Alcohol consumption | 24 (1.1) | 76 (0.5) | 100 (0.6) |
| Opiates intake | 2 (0.1) | 22 (0.1) | 24 (0.1) |
|  |  |  |  |

# **Table S2**. Variables’ definition

|  |  |  |
| --- | --- | --- |
| **Variables** | **Identification criteria: ICD-10 diagnosis codes, exclusion criteria and medical procedures when necessary** | **French medical classification for clinical procedures codes (CCAM) used for medical procedures, surgical repair, and imaging** |
|  |  |  |
| **Previous serious infections** |  |  |
| **Bacterial infections** | A00-A05, A403, A20–A28, A32-A40, A410-A415, A42-A57, A65–A79, B950-B958, B96–B97, G00-G01, G042, G050, H000, H010, H105, H600–H603,H620, H651, H66, H670, H70, H750, I320, I410, I520, I980, J020, J030, J051,J13-J15, J160, J170, J200-J202, J340, J36, J390-J391, K113, K122, K670-K672, K800-K801, K803-K804, K810, K830, L00-L03, L05, L080-L081, M00,M010, M013, M491-M492, M630, M726, N136, N300, N410, N412, N413,P36, P372, P38 |  |
| **Viral infections** | A08, A60, A80–A99, B00–B02, B05-B06, B08-B09, B15-B27, B30, B33–B34,G020, G041, G051, H191, H621, H671, I411, J050, J09-J12, J171, J203-J207,J21, K2380, K8700, K93820, M014-M015, P35 |  |
| **Fungal infections** | B37-B40, B44-B45, B59, G021, J172 |  |
| **Bronchopulmonary dysplasia** | P271 |  |
| **Cystic fibrosis** | E84 |  |
| **Neonatal respiratory distress** |  |  |
| Meconium aspiration syndrome | P240 |  |
| Pneumothorax | P251 |  |
| Respiratory distress syndrome | P22 |  |
| **Congenital heart defects and diseases** |  |  |
| **Congenital heart diseases** |  |  |
| Pulmonary hypertension | I270 |  |
| Other secondary pulmonary hypertension | I272 |  |
| Unspecified congenital malformations of heart | Q248/Q249 |  |
| **Congenital heart defects** |  |  |
| Common arterial trunk | Q200 |  |
| Double outlet right ventricle | Q201 |  |
| Double outlet left ventricle | Q202 |  |
| Discordant ventriculoarterial connection | Q203 |  |
| Discordant atrioventricular connection | Q205 + surgical repair within 1 year OR death | DZMA010, DZMA001, DZMA002, DZMA003, DZMA004, DZMA006 |
| Double inlet ventricle | Q204, exclude if associated with Hypoplastic left heart syndrome (Q234) or Hypoplastic right heart syndrome (Q226) |  |
| Ventricular septal defect | Q210 OU surgical repair within 1 year | DASA001, DASA004, DASA006, DASA007, DASA009, DASA010, DASA011, DASA012, DASA014, DASF003, DFGA002, DFMA011, DZMA004, DZMA001, DZMA002, DZMA003, DZMA010, DZMA006, DFGA004, DFMA012 |
|  |  |  |
| Atrial septal defect, incl. persistent foramen ovale | Q211 + at least one echography within 1 year OR death | DZQJ001, DZQJ006, DZQJ008, DZQJ009, DZQJ010, DZQJ011, DZQM005, DZQM006 |
| Atrioventricular septal defect | Q212 |  |
| Tetralogy of Fallot | Q213 |  |
| Congenital tricuspid stenosis | Q224 |  |
| Ebstein anomaly | Q225 |  |
| Congenital pulmonary valve stenosis | Q221 |  |
| Pulmonary valve atresia | Q220 |  |
| Congenital stenosis of aortic valve | Q230 |  |
| Congenital mitral stenosis | Q232 + surgical repair within 1 year OR death | DBMA002, DBMA003 |
| Hypoplastic left heart syndrome | Q234 |  |
| Hypoplastic right heart syndrome | Q226 |  |
| Coarctation of aorta | Q251 + medical procedure within 1 year OR death | EQLF005, DGFA020, DGAF004, DGAF006, DGAA005, DGAA004, DGAA006, DGAA003, DGAA002, DGKA017, DGKA021, DGKA022, DGKA024 |
| Atresia of aorta | Q252 |  |
| Total anomalous pulmonary venous connection | Q262 |  |
| Patent ductus arteriosus, only in terms infants (gestational age > 37 weeks) | Q250 + surgical closure within 1 year | DASF001 |
|  | OR Q25O still present after 6 months OR death |  |
|  | AND not part of a ductus dependent congenital heart defects, namely: transposition of great arteries (Q203), hypoplastic left heart (Q234) and Coarctation of aorta (Q251) |  |
| **Congenital anomalies of the nervous system and chromosomal abnormalities** |  |  |
| **Anomalies of the nervous system** |  |  |
| Anencephaly and similar malformations | Q00 |  |
| Encephalocele | Q01, exclude if associated with Anencephaly (Q00) |  |
| Spina Bifida | Q05, exclude if associated with Anencephaly (Q00) or Encephalocele (Q01) |  |
| Congenital hydrocephalus | Q03, exclude if associated with Neural Tube defect group (Q00, Q01, Q05) |  |
| Microcephaly | Q02, exclude if associated with Neural Tube defect group (Q00, Q01, Q05) + at least 1 MRI within 1 or 2 year(s) or death |  |
| Arhinencephaly/Holoprosencephaly | Q041, Q042, exclude if associated with Neural Tube defect group (Q00, Q01, Q05) |  |
| Congenital malformations of corpus callosum | Q040, exclude if associated with Neural Tube defect group (Q00, Q01, Q05) |  |
| **Chromosomal** |  |  |
| Skeletal dysplasia | Q77, Q780-Q788 |  |
| Down syndrome | Q90 |  |
| Trisomy 13/Patau syndrome | Q914-Q917 |  |
| Trisomy 18/Edwards syndrome | Q910-Q913 |  |
| Turner syndrome | Q96 |  |
| Triploidy and polyploidy | Q927 |  |
| **Respiratory and esophageal abnormalities** |  |  |
| Atresia of oesophagus with/without tracheo-oesophageal fistula | Q390, Q391 |  |
| Congenital diaphragmatic hernia | Q790 |  |
| **Respiratory anomalies** |  |  |
| Choanal atresia | Q300+ surgical repair within 1 year or death | GCME004, GCME003, GCME002, GCMA001, GCME001, GCCD001 |
| **Other malformations or non-specific anomalies** |  |  |
| **Anomalies of the eyes** |  |  |
| Cystic eyeball/Other anophthalmos/Microphthalmos | Q110, Q111, Q112 |  |
| Cystic eyeball/Other anophthalmos | Q110, Q111 |  |
| Congenital cataract | Q120 + specific medical procedures within 1 year or death | BFPA002, BGFA008, BFGA002, BGFA001, BFGA008 |
| Congenital glaucoma | Q150 + specific medical procedures within 1 year or death | BHQP002, BEFA008, BEPA003, BGFA014 |
| **Anomalies of the ear, face and neck** |  |  |
| Congenital absence of (ear) auricle/Congenital absence atresia and structure of auditory canal (external) | Q160, Q161 |  |
| **Oro-facial clefts** |  |  |
| Cleft palate | Q35 exclude Q357 and exclude if associated with holoprosencephaly subgroup, or cleft lip subgroups |  |
| Cleft lip/Cleft palate with cleft lip | Q36, Q37 and exclude if associated with holoprosencephaly subgroup |  |
| **Anomalies of the digestive system** |  |  |
| Congenital absence, atresia and stenosis of duodenum | Q410, exclude if associated with annular pancreas subgroup |  |
| Congenital absence, atresia and stenosis of jejunum/ileum/ other specified parts of small intestine | Q411-Q418 |  |
| Congenital absence, atresia and stenosis of anus/rectum with/without fistula | Q420-Q423 + surgical repair within 1 year or death | HJAD001, HJEA001, HJEA002, HJEA003, HJEA004, HHCA002, HJMA001, HKEA001, HKMA006, HHCC007 |
| Hirschprung disease | Q431 + surgical repair within 1 year or death | HJFD003, HJFC001, HJFA016, HHCA002, HHCC007 |
| Congenital malformations of intestinal fixation | Q433 |  |
| Atresia of bile ducts | Q442 + surgical repair within 1 year or death | HLCA001 |
| Annular pancreas | Q451 |  |
| **Abdominal wall defects** | | |
| Gastroschisis | Q793 |  |
| Exomphalos | Q792 |  |
| **Congenital anomalies of kidney and urinary tract** | | |
| Renal agenesis, unilateral | Q600 |  |
| Renal agenesis, bilateral/ Potter syndrome | Q601, Q606 |  |
| Renal dysplasia | Q614 |  |
| Congenital hydronephrosis/Atresia and stenosis of ureter/Other obstructive defects of renal pelvis and ureter | Q620, Q621, Q623 exclude if associated with Q627 + at least 2 echographies within 1 year or death | JAQM003, JAQM004, JAQJ001, JAQM001 |
| Lobulated, fused and horseshoe kidney/Ectopic kidney | Q631, Q632 |  |
| Epispadias/ Exstrophy of urinary bladder | Q640 + surgical repair ( JHAA001, JHAA002) within 1 or 2 year(s) or death | JHAA001, JHAA002 |
|  | Q641 + surgical repair ( JDS001, JDSA003, JDS004, JDSA007, JDSA009, JDFA010, JDFA012, JDFA013) within 1 or death | JDS001, JDSA003, JDS004, JDSA007, JDSA009, JDFA010, JDFA012, JDFA013 |
| Congenital posterior urethral valves | Q642 + surgical repair within 1 year or death | JEFE005, JEPH001 |
| **Genital** |  |  |
| Prune belly syndrome | Q794 |  |
| Hypospadias | Q54 excluding Q544 + surgical repair within 1 or 2 years | JEMA006, JEMA014, JEMA019, JEMA020, JEMA021, JEMA011 |
| Indeterminate sex and pseudohermaphroditism | Q56 |  |
| **Limb anomalies** |  |  |
| Reduction defects of upper/lower/unspecified limb | Q71, Q72, Q73 |  |
| Talipes equinovarus | Q660 + specific medical procedures within 1 year OR death | NHRP003, NJAB001, PCPB002 |
| Congenital dislocation of hip, unilateral/bilateral/unspecified | Q650, Q651, Q652 + surgical repair within 1 year OR at least 2 diagnostic tests within 1 year OR death | Surgery: NEEP003, NEEA004, ZEMP002, NEQP001, NEQH001, NEEP006, NEQP002, NZMP012, ZEMP010 |
|  |  | diagnostic test: NEQM001, NEQH002, NAQK071, NEQC001 |
| Polydactyly | Q69 + surgical repair within 1 year OR death | MZFA008, MZFA012, MZFA015, MZFA014, NZFA011, NZFA012 |
| Syndactyly | Q70 + surgical repair within 1 year OR death | MJPA014, MZPA002, QDPA001 |
| **Other anomalies** |  |  |
| Craniosynostosis | Q750 + surgical repair within 1 year or death | LAFA900, LAMA006, LANC001, LAPA005, LAPA006, LAPA008, LAPA016, LAEA002, LAEA004, LAEA006, LAEA009, LARA001, LARA002, LARA003, LARA004 |
| Situs inversus | Q893 |  |
| Septo-optic dysplasia | Q044 |  |
| Vascular disruption anomalies | Q411, Q412, Q418, Q710, Q712, Q720, Q722, Q730, Q793 |  |
| Laterality anomalies | Q206, Q240, Q890, Q893 |  |
| Maternal infections | P350, P351, P354, P358, P371 |  |
| Teratogenic syndromes resulting in major malformation | Q86, P350, P351, P354, P358, P371 |  |
|  |  |  |

# **Table S3.** Parameter estimates from the propensity score analysis

| Parameter | Estimate | Standard Error | Pr>ChiSq |
| --- | --- | --- | --- |
| Intercept | 2,1509 | 0,8758 | 0,014 |
| Sex (ref=male) | 0,00168 | 0,0122 | 0,8904 |
| Gestational age | -0,0564 | 0,0106 | <.0001 |
| Month of birth (ref=October) |  |  |  |
| September | -0,2051 | 0,3538 | 0,5621 |
| November | 0,0725 | 0,1193 | 0,5438 |
| December | 0,1223 | 0,1192 | 0,3046 |
| Region (ref=Provence-Alpes-Côte d’Azur) |  |  |  |
| Auvergne-Rhône-Alpes | 0,0332 | 0,0361 | 0,3574 |
| Bourgogne-Franche-Comté | -0,4699 | 0,0556 | <.0001 |
| Bretagne | 0,9436 | 0,0571 | <.0001 |
| Centre-Val de Loire | 0,1003 | 0,0569 | 0,0779 |
| Corse | -0,8883 | 0,1733 | <.0001 |
| Grand Est | -0,3154 | 0,044 | <.0001 |
| Hauts-de-France | 0,381 | 0,041 | <.0001 |
| Ile-de-France | -0,7661 | 0,0329 | <.0001 |
| Normandie | 0,8335 | 0,0582 | <.0001 |
| Nouvelle-Aquitaine | 0,3318 | 0,0473 | <.0001 |
| Occitanie | 0,1483 | 0,0478 | 0,0019 |
| Pays de la Loire | 0,4473 | 0,0556 | <.0001 |
| Birth weight (ref = Appropriate (P10-P90)) |  |  |  |
| Small (<P10) | 0,0956 | 0,2567 | 0,7096 |
| Large (>P90) | -0,0255 | 0,2567 | 0,921 |
| Missing | -0,138 | 0,7652 | 0,8569 |
| Previous serious infections (ref = No) | -0,1897 | 0,0611 | 0,0019 |
| Cystic fibrosis (ref = No) | 1,1592 | 0,5485 | 0,0346 |
| Neonatal respiratory distress (ref = No) | 0,0509 | 0,0323 | 0,1146 |
| Congenital heart defects and diseases (ref = No) | 0,05 | 0,1568 | 0,7499 |
| Congenital anomalies of the nervous system and chromosomal abnormalities (ref = No) | -0,0495 | 0,1908 | 0,7954 |
| Respiratory and oesophageal abnormalities (ref = No) | -0,2038 | 0,3842 | 0,5959 |
| Other malformations or non-specific anomalies (ref = No) | 0,0575 | 0,091 | 0,5269 |
| Mode of delivery (ref=vaginal) |  |  |  |
| Caesarean | 0,0462 | 0,0572 | 0,4197 |
| Missing | -0,101 | 0,1081 | 0,3502 |
| Deprivation index (ref=Q3 / Q4 / Q5 (more deprived)) |  |  |  |
| Q1 (less deprived) / Q2 | 0,1805 | 0,1441 | 0,2101 |
| Missing | -0,2612 | 0,2866 | 0,362 |
| General Practitioners’ Localized Potential — Quartiles (ref=>4.7) |  |  |  |
| <=3.0 | -0,1437 | 0,0218 | <.0001 |
| 3.0-3.8 | -0,1125 | 0,021 | <.0001 |
| 3.8-4.7 | 0,1697 | 0,021 | <.0001 |
| Complementary solidarity health insurance status (ref= No) | -0,4575 | 0,0178 | <.0001 |
| Maternal and child welfare centres (ref= No) | -0,0759 | 0,0266 | 0,0043 |
| Social security affiliation type (ref=General health scheme) |  |  |  |
| Agricultural scheme | -0,022 | 0,0629 | 0,7258 |
| Other | 0,0982 | 0,0785 | 0,2111 |
| Maternal age at childbirth – years (ref=15-24) |  |  |  |
| 25-29 | -0,0908 | 0,0213 | <.0001 |
| 30-34 | 0,2384 | 0,0201 | <.0001 |
| 35-49 | 0,3112 | 0,0225 | <.0001 |
| Maternal Parity (ref=1) |  |  |  |
| 2 | 0,261 | 0,023 | <.0001 |
| 3 | -0,2126 | 0,0306 | <.0001 |
| ≥ 4 | -0,6153 | 0,0435 | <.0001 |
| Tobacco use | 0,07 | 0,0199 | 0,0004 |
| Alcohol consumption | -0,1047 | 0,0846 | 0,2163 |
|  |  |  |  |

# **Table S4.** Extensive Infants and maternal characteristics at inclusion

|  | **Unimmunised** | **RSVpreF vaccine** | **Total** |
| --- | --- | --- | --- |
|  | **(N = 15 678)** | **(N = 15 678)** | **(N = 31 376)** |
|  |  |  |  |
| ***Characteristics*** |  |  |  |
|  |  |  |  |
| **Gestation at RSVpreF vaccine injection — wk.** |  |  |  |
| Mean | - | 34.6 (1.7) | - |
| Median (range) | - | 34.9 (33.6-35.7) | - |
| within 32–36 weeks | - | 13317 (84.9) | - |
|  |  |  |  |
| **Time between mother's vaccination and discharge from birth hospitalization — wk.** | |  |  |
| Mean | - | 5.2 (1.7) | - |
| Median (IQR) | - | 5.0 (3.9-6.1) | - |
|  |  |  |  |
| ***Comorbidities*** |  |  |  |
|  |  |  |  |
| **Cystic fibrosis — no. of persons (%)** | 1 (0.0) | 8 (0.1) | 9 (0.0) |
|  |  |  |  |
| **Neonatal respiratory distress — no. of persons (%)** | 581 (3.7) | 636 (4.1) | 1217 (3.9) |
| Meconium aspiration syndrome | 81 (0.5) | 67 (0.4) | 148 (0.5) |
| Pneumothorax | 7 (0.0) | 32 (0.2) | 39 (0.1) |
| Respiratory distress syndrome | 534 (3.4) | 605 (3.9) | 1139 (3.6) |
|  |  |  |  |
| **Major congenital malformation — no. of persons (%)** | 146 (0.9) | 161 (1.0) | 307 (1.0) |
|  |  |  |  |
| **Congenital heart defects and diseases — no. of persons (%)** | 75 (0.5) | 71 (0.5) | 146 (0.5) |
| Congenital heart defects | 58 (0.4) | 55 (0.4) | 113 (0.4) |
| Common arterial trunk | 0 (0.0) | 1 (0.0) | 1 (0.0) |
| Double outlet right ventricle | 4 (0.0) | 1 (0.0) | 5 (0.0) |
| Double outlet left ventricle | 0 (0.0) | 0 (0.0) | 0 (0.0) |
| Discordant ventriculoarterial connection | 10 (0.1) | 5 (0.0) | 15 (0.0) |
| Discordant atrioventricular connection | 1 (0.0) |  | 1 (0.0) |
| Double inlet ventricle | 3 (0.0) | 1 (0.0) | 4 (0.0) |
| Ventricular septal defect | 28 (0.2) | 23 (0.1) | 51 (0.2) |
| Atrial septal defect, incl. persistent foramen ovale | 17 (0.1) | 16 (0.1) | 33 (0.1) |
| Atrioventricular septal defect | 2 (0.0) | 3 (0.0) | 5 (0.0) |
| Tetralogy of Fallot | 2 (0.0) | 3 (0.0) | 5 (0.0) |
| Congenital tricuspid stenosis | 0 (0.0) | 1 (0.0) | 1 (0.0) |
| Ebstein anomaly | 2 (0.0) | 0 (0.0) | 2 (0.0) |
| Congenital pulmonary valve stenosis | 3 (0.0) | 3 (0.0) | 6 (0.0) |
| Pulmonary valve atresia | 0 (0.0) | 2 (0.0) | 2 (0.0) |
| Congenital stenosis of aortic valve | 0 (0.0) | 1 (0.0) | 1 (0.0) |
| Congenital mitral stenosis | 0 (0.0) | 0 (0.0) | 0 (0.0) |
| Hypoplastic left heart syndrome | 4 (0.0) | 5 (0.0) | 9 (0.0) |
| Hypoplastic right heart syndrome | 1 (0.0) | 2 (0.0) | 3 (0.0) |
| Coarctation of aorta | 5 (0.0) | 4 (0.0) | 9 (0.0) |
| Atresia of aorta | 0 (0.0) | 0 (0.0) | 0 (0.0) |
| Total anomalous pulmonary venous connection | 1 (0.0) | 1 (0.0) | 2 (0.0) |
| Congenital heart diseases | 25 (0.2) | 26 (0.2) | 51 (0.2) |
| Pulmonary hypertension | 7 (0.0) | 6 (0.0) | 13 (0.0) |
| Other secondary pulmonary hypertension | 11 (0.1) | 9 (0.1) | 20 (0.1) |
| Unspecified congenital malformations of heart | 11 (0.1) | 14 (0.1) | 25 (0.1) |
|  |  |  |  |
| **Congenital anomalies of the nervous system and chromosomal abnormalities — no. of persons (%)** | 16 (0.1) | 16 (0.1) | 32 (0.1) |
| Congenital anomalies of the nervous system |  |  |  |
| Anencephaly and similar malformations | 0 (0.0) | 0 (0.0) | 0 (0.0) |
| Congenital hydrocephalus | 3 (0.0) | 1 (0.0) | 4 (0.0) |
| Microcephaly | 1 (0.0) | 0 (0.0) | 1 (0.0) |
| Encephalocele | 0 (0.0) | 3 (0.0) | 3 (0.0) |
| Arhinencephaly/Holoprosencephaly | 0 (0.0) | 0 (0.0) | 0 (0.0) |
| Congenital malformations of corpus callosum | 3 (0.0) | 2 (0.0) | 5 (0.0) |
| Spina Bifida | 3 (0.0) | 2 (0.0) | 5 (0.0) |
| Chromosomal abnormalities |  |  |  |
| Dysplasia of the septum and optic pathways | 1 (0.0) | 1 (0.0) | 2 (0.0) |
| Skeletal dysplasia | 1 (0.0) | 0 (0.0) | 1 (0.0) |
| Down syndrome | 2 (0.0) | 7 (0.0) | 9 (0.0) |
| Trisomy 13/Patau syndrome | 0 (0.0) | 0 (0.0) | 0 (0.0) |
| Trisomy 18/Edwards syndrome | 2 (0.0) | 0 (0.0) | 2 (0.0) |
|  |  |  |  |
| **Respiratory and oesophageal abnormalities — no. of persons (%)** | 5 (0.0) | 3 (0.0) | 8 (0.0) |
| Choanal atresia | 0 (0.0) | 0 (0.0) | 0 (0.0) |
| Congenital diaphragmatic hernia | 4 (0.0) | 0 (0.0) | 4 (0.0) |
| Atresia of oesophagus with/without trachea-oesophageal fistula | 1 (0.0) | 3 (0.0) | 4 (0.0) |
|  |  |  |  |
| **Other malformations or non-specific anomalies — no. of persons (%)** | 65 (0.4) | 80 (0.5) | 145 (0.5) |
| Anomalies of the digestive system |  |  |  |
| Congenital absence, atresia and stenosis of duodenum | 3 (0.0) | 0 (0.0) | 3 (0.0) |
| Congenital absence, atresia and stenosis of jejunum/ileum/ other specified parts of small intestine | 1 (0.0) | 0 (0.0) | 1 (0.0) |
| Congenital absence, atresia and stenosis of anus/rectum with/without fistula | 3 (0.0) | 3 (0.0) | 6 (0.0) |
| Hirschprung disease | 1 (0.0) | 1 (0.0) | 2 (0.0) |
| Congenital malformations of intestinal fixation | 0 (0.0) | 0 (0.0) | 0 (0.0) |
| Atresia of bile ducts | 0 (0.0) | 0 (0.0) | 0 (0.0) |
| Annular pancreas | 0 (0.0) | 0 (0.0) | 0 (0.0) |
| Abdominal wall defects |  |  |  |
| Gastroschisis | 0 (0.0) | 0 (0.0) | 0 (0.0) |
| Exomphalos | 5 (0.0) | 4 (0.0) | 9 (0.0) |
| Congenital anomalies of kidney and urinary tract |  |  |  |
| Renal agenesis, unilateral | 4 (0.0) | 11 (0.1) | 15 (0.0) |
| Renal agenesis, bilateral/ Potter syndrome | 4 (0.0) | 11 (0.1) | 15 (0.0) |
| Renal dysplasia | 1 (0.0) | 5 (0.0) | 6 (0.0) |
| Congenital hydronephrosis/Atresia and stenosis of ureter/Other obstructive defects of renal pelvis and ureter | 4 (0.0) | 7 (0.0) | 11 (0.0) |
| Lobulated, fused and horseshoe kidney/Ectopic kidney | 10 (0.1) | 3 (0.0) | 13 (0.0) |
| Epispadias/ Exstrophy of urinary bladder | 0 (0.0) | 0 (0.0) | 0 (0.0) |
| Congenital posterior urethral valves | 2 (0.0) | 0 (0.0) | 2 (0.0) |
| Genital malformations |  |  |  |
| Prune belly syndrome | 0 (0.0) | 0 (0.0) | 0 (0.0) |
| Hypospadias | 1 (0.0) | 0 (0.0) | 1 (0.0) |
| Indeterminate sex and pseudohermaphrodites | 2 (0.0) | 0 (0.0) | 2 (0.0) |
| Limb anomalies |  |  |  |
| Reduction defects of upper/lower/unspecified limb | 2 (0.0) | 2 (0.0) | 4 (0.0) |
| Talipes equinovarus | 8 (0.1) | 6 (0.0) | 14 (0.0) |
| Congenital dislocation of hip, unilateral/bilateral/unspecified | 0 (0.0) | 7 (0.0) | 7 (0.0) |
| Polydactyly | 2 (0.0) | 2 (0.0) | 4 (0.0) |
| Syndactyly | 0 (0.0) | 0 (0.0) | 0 (0.0) |
| Other anomalies |  |  |  |
| Maternal infections | 8 (0.1) | 11 (0.1) | 19 (0.1) |
| Teratogenic syndromes resulting in major malformation | 8 (0.1) | 11 (0.1) | 19 (0.1) |
| Anomalies of the ear, face and neck |  |  |  |
| Congenital absence of (ear) auricle/Congenital absence atresia and structure of auditory canal (external) | 0 (0.0) | 1 (0.0) | 1 (0.0) |
| Oro-facial clefts | 3 (0.0) | 0 (0.0) | 3 (0.0) |
| Cleft palate | 2 (0.0) | 3 (0.0) | 5 (0.0) |
| Cleft lip/Cleft palate with cleft lip | 5 (0.0) | 14 (0.1) | 19 (0.1) |
|  |  |  |  |

# **Table S5.** Sensitivity analysis for the effectiveness of the maternal RSVpreF vaccination against RSV-associated hospitalization

|  | **Unimmunized** | | **RSVpreF vaccine** | | **wHR (RSVpreF vs. unvaccinated)** | **Effectiveness** |
| --- | --- | --- | --- | --- | --- | --- |
| **Hospitalization for RSV-associated lower respiratory tract infection (RSV-LRTI)** | No. Events / No. Participants (%) | Median follow up (days) | No. Events / No. Participants (%) | Median follow up (days) | 95% (CI) | 95% (CI) |
|  |  |  |  |  |  |  |
| *Sensitivity analyses* |  |  |  |  |  |  |
|  |  |  |  |  |  |  |
| **Alternative statistical computations** |  |  |  |  |  |  |
| Multivariate Cox model | 477 / 15 678 (3.0) | 86 [73-101] | 216 / 15 678 (1.4) | 86 [73-101] | 0.51 [0.46-0.57] | 49% |
| Propensity score with trimming (1%) | 446 / 15 058 (3.0) | 86 [73-101] | 211 / 15 058 (1.4) | 86 [73-101] | 0.52 [0.47-0.57] | 48% |
| Restriction to a single use of each unimmunised infant | 370 / 11 431 (3.2) | 90 [74-106] | 159 / 11 431 (1.4) | 90 [74-106] | 0.49 [0.45-0.53] | 51% |
|  |  |  |  |  |  |  |
| **Time between RSVpreF vaccine and delivery — d.** |  |  |  |  |  |  |
|  |  |  |  |  |  |  |
| Infants born 14-30 days after maternal vaccination | 185 / 5 604 (3.3) | 92 [76-109] | 89 / 5 604 (1.6) | 92 [76-109] | 0.53 [0.46-0.60] | 47% |
| Infants born > 30 days after maternal vaccination | 292 / 10 074 (2.9) | 83 [71-98] | 127 / 10 074 (1.3) | 83 [71-98] | 0.48 [0.43-0.54] | 52% |
|  |  |  |  |  |  |  |
| **Time between RSVpreF vaccine and delivery — wk.** |  |  |  |  |  |  |
|  |  |  |  |  |  |  |
| < 6 weeks | 342 / 10 988 (3.1) | 89 (74-105) | 167 / 10 988 (1.5) | 89 (74-105) | 0.54 [0.49-0.59] | 46% |
| ≥ 6 weeks | 135 / 4 690 (2.9) | 80 (70-93) | 49 / 4 690 (1.0) | 80 (70-93) | 0.40 [0.34-0.47] | 60% |
|  |  |  |  |  |  |  |


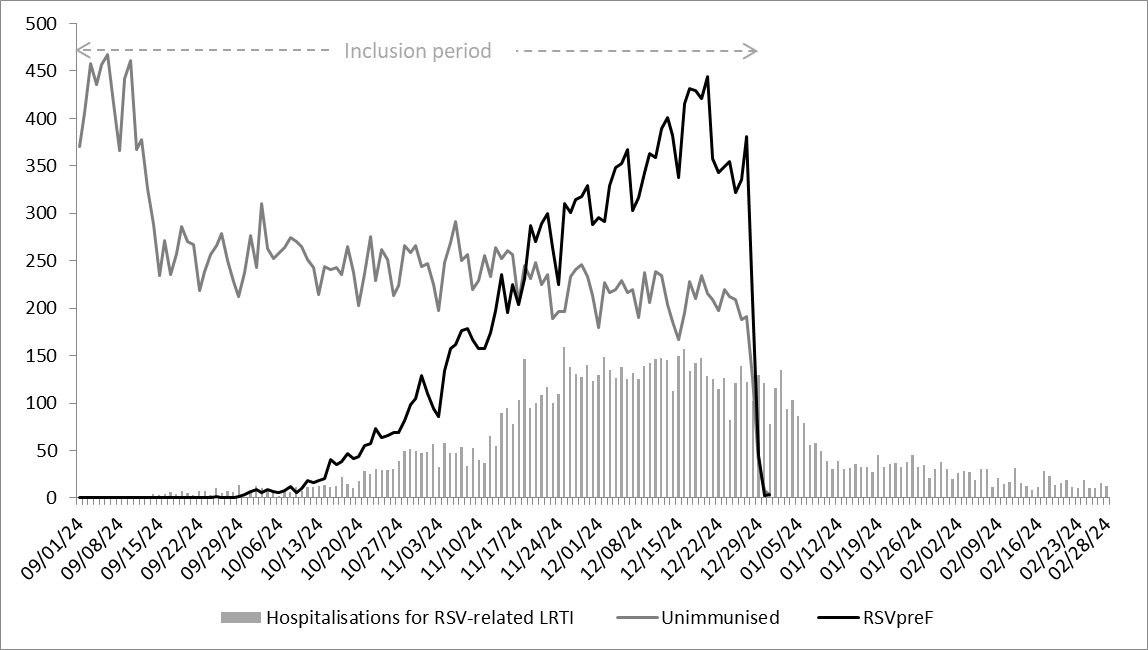


# **Figure S1.** Temporal trends in RSV-related infant hospitalisations in France during the 2024–2025 season among all infants born in 2024 (N = 650,000), and daily numbers of infants born to mothers immunised with RSVpreF and to unimmunised mothers from Sept 1 to Dec 31, 2024.


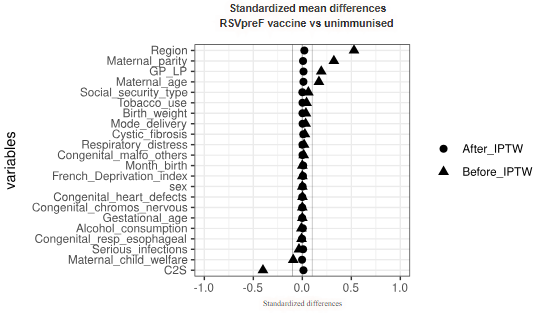


# **Figure S2.** Standardized mean differences before and after inverse probability of treatment weighting

# **
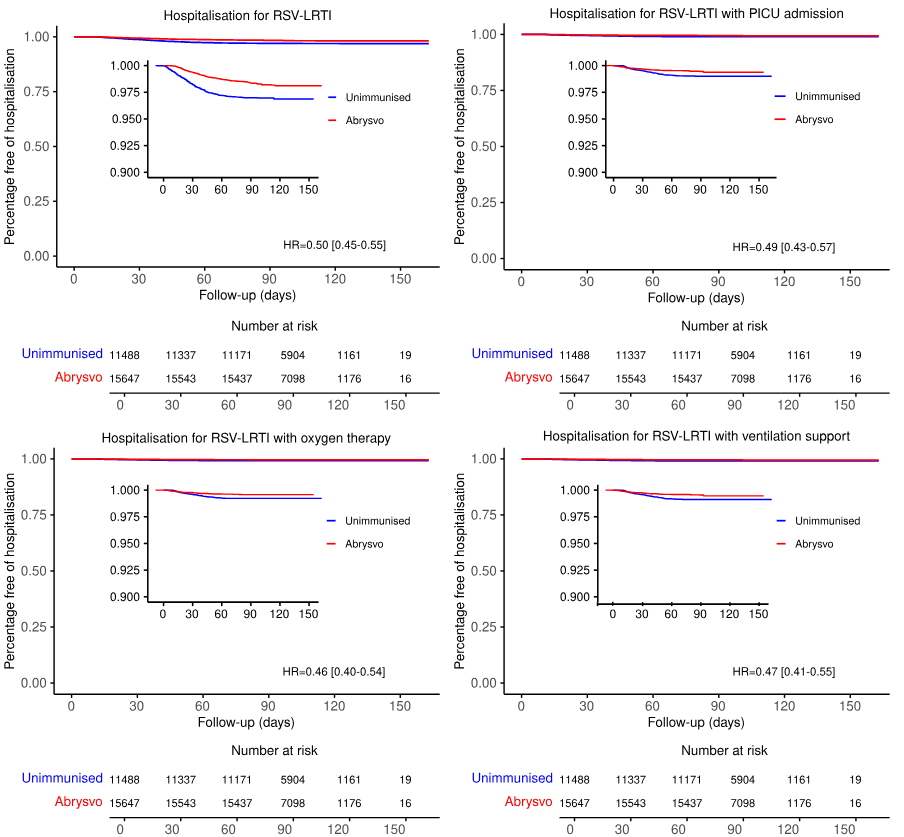
**

# **Figure S3.** Hospitalization for RSV-related lower respiratory tract infection (RSV-LRTI) by group using IPTW Kaplan-Meier curves

# **Appendix Methods.** Specification of the Target Trial and Its Emulation

| **Component** | **Target Trial Specification** | **Emulation Using the SNDS** |
| --- | --- | --- |
| **Eligibility criteria** | Infants entering their first RSV season whose mothers are eligible to receive maternal RSVpreF vaccination during pregnancy. | Live-born infants recorded in the SNDS between Sept 1 and Dec 31, 2024 in mainland France, born to mothers aged 15–50 years and linked to their mother. Infants with missing gestational age or born within 14 days after maternal vaccination were excluded. |
| **Treatment strategies** | Strategy 1: maternal RSVpreF vaccination during pregnancy. Strategy 2: no maternal RSV immunisation during pregnancy. | Exposure defined using RSVpreF vaccine dispensing during pregnancy identified by ATC code J07BX05 in the SNDS. |
| **Assignment procedure** | Random allocation to vaccination or no vaccination at eligibility. | 1:1 exact matching on date of birth, gestational age, sex, and deprivation status, followed by inverse probability of treatment weighting based on baseline covariates. |
| **Start of follow-up (time zero)** | Birth of the infant. | Follow-up started at birth, aligning eligibility, exposure assignment, and follow-up initiation to avoid immortal time bias. |
| **Outcome** | RSV-associated lower respiratory tract infection hospitalisation during the first RSV season. | Hospitalisation identified using ICD-10 codes for RSV-LRTI (J210, J121, J205) in the PMSI hospital database. |
| **Follow-up** | From birth until RSV hospitalisation, death, or end of the RSV season. | From birth until RSV-LRTI hospitalisation, death, or Feb 28, 2025. |
| **Causal contrast** | Intention-to-treat effect of maternal vaccination versus no vaccination. | Hazard ratio estimated using weighted Cox proportional hazards models. Vaccine effectiveness calculated as (1-HR)×100. |
| **Sensitivity analyses** | Assessment of robustness to alternative analytic assumptions. | Analyses trimming extreme weights, multivariable adjustment without weighting, and restricting controls to a single match. |
